# Metagenomic Study Suggests That the Gut Microbiota of the Giant Panda (*Ailuropoda melanoleuca*) May Not Be Specialized for Fiber Fermentation

**DOI:** 10.3389/fmicb.2018.00229

**Published:** 2018-02-16

**Authors:** Wei Guo, Sudhanshu Mishra, Jiangchao Zhao, Jingsi Tang, Bo Zeng, Fanli Kong, Ruihong Ning, Miao Li, Hengzhi Zhang, Yutian Zeng, Yuanliangzi Tian, Yihang Zhong, Hongdi Luo, Yunhan Liu, Jiandong Yang, Mingyao Yang, Mingwang Zhang, Yan Li, Qingyong Ni, Caiwu Li, Chengdong Wang, Desheng Li, Hemin Zhang, Zhili Zuo, Ying Li

**Affiliations:** ^1^Farm Animal Genetic Resources Exploration and Innovation Key Laboratory of Sichuan Province, Sichuan Agricultural University, Chengdu, China; ^2^Division of Agriculture, Department of Animal Science, University of Arkansas, Fayetteville, AR, United States; ^3^Animal Nutrition Institute, Sichuan Agricultural University, Chengdu, China; ^4^College of Animal Science and Technology, Sichuan Agricultural University, Ya'an, China; ^5^China Conservation and Research Center for the Giant Panda, Ya'an, China; ^6^Chengdu Zoo, Chengdu Institute of Wildlife, Chengdu, China

**Keywords:** giant panda, gut microbiota, metagenomics, enzyme activity, diet adaptability

## Abstract

Bamboo-eating giant panda (*Ailuropoda melanoleuca*) is an enigmatic species, which possesses a carnivore-like short and simple gastrointestinal tract (GIT). Despite the remarkable studies on giant panda, its diet adaptability status continues to be a matter of debate. To resolve this puzzle, we investigated the functional potential of the giant panda gut microbiome using shotgun metagenomic sequencing of fecal samples. We also compared our data with similar data from other animal species representing herbivores, carnivores, and omnivores from current and earlier studies. We found that the giant panda hosts a bear-like gut microbiota distinct from those of herbivores indicated by the metabolic potential of the microbiome in the gut of giant pandas and other mammals. Furthermore, the relative abundance of genes involved in cellulose- and hemicellulose-digestion, and enrichment of enzymes associated with pathways of amino acid degradation and biosynthetic reactions in giant pandas echoed a carnivore-like microbiome. Most significantly, the enzyme assay of the giant panda's feces indicated the lowest cellulase and xylanase activity among major herbivores, shown by an *in-vitro* experimental assay of enzyme activity for cellulose and hemicellulose-degradation. All of our results consistently indicate that the giant panda is not specialized to digest cellulose and hemicellulose from its bamboo diet, making the giant panda a good mammalian model to study the unusual link between the gut microbiome and diet. The increased food intake of the giant pandas might be a strategy to compensate for the gut microbiome functions, highlighting a strong need of conservation of the native bamboo forest both in high- and low-altitude ranges to meet the great demand of bamboo diet of giant pandas.

## Introduction

The giant panda (*Ailuropoda melanoleuca*) is a highly vulnerable mammal (The IUCN Red List of Threatened Species, 2016) while being a flagship species for wildlife conservation worldwide (Wei et al., 2012, 2015a). Phylogenetically, the giant panda belongs to the family Ursidae, which includes both carnivorous and omnivorous members (Arnason et al., 2007; Krause et al., 2008). This enigmatic species is of great interest for researchers and conservationists because it consumes large quantities of highly fibrous bamboo leaves or stems each day, despite the species possessing a typical carnivore like short and simple gastrointestinal tract (GIT) (Dierenfeld et al., 1982; Schaller et al., 1985). Moreover, along with the threat of climate change (Li et al., 2015a; Shen et al., 2015), this bamboo specialist is at risk of extinction because of its sole dependency on large quantities of low-nutrition and low-energy content food from a limited number of plant species (Dierenfeld et al., 1982; Colles et al., 2009).

In view of this, different facets of the giant panda were researched to know the plight of its future survival. Adaptive cranial anatomy of the skull indicates that the early omnivorous giant pandas became full-time bamboo-eating species by the late Pliocene (2 Mya) (Jin et al., 2007). The giant pandas have strong skull structure with dense compact bones and well-developed mandible (Sicher, 1944; Davis, 1964; Zhang et al., 2007), extensive jaw muscles, flattened molars (Davis, 1964; Owen, 1980; Eisenberg, 1981), and evolved “pseudothumb: an enlarged sesamoid bone” (Endo et al., 1999; Salesa et al., 2006) which increases foraging efficacy (Endo et al., 1999). Furthermore, giant pandas display unique seasonal foraging behaviors in terms of preferring bamboo species (Schaller et al., 1985; Liu et al., 2005; Tarou et al., 2005) and selecting plant parts (leaves and culms) (Hansen et al., 2010; Williams et al., 2016) to maximize energetic gains. Studies have reported that Tas1r1 (umami taste receptor gene) was pseudogenized in the giant panda, which occurred about 4.2 Mya, possibly contributing to its dietary switch (Li et al., 2010; Zhao et al., 2010). Additionally, other factors may also be involved since horse and cow still have an intact Tas1r1 gene (Li et al., 2010; Zhao et al., 2010). Besides, an incompetent dopamine metabolism in the “appetite-reward system” in the panda genome was believed to be a governing factor in the food switch of this species (Jin et al., 2011). But this finding was disapproved by a recent study (Tang et al., 2015). Despite these excellent studies on the evolutionary consequences of the dietary switch of the pandas, there is still uncertainty about the underlying mechanisms, and one question remains unanswered; “Why did pandas shift to bamboo diet?” (Wei et al., 2012). Also, the giant pandas have not evolved adaptations like an enlarged cecum or a rumen, to degrade fiber, which is typically present in common herbivores (Davis, 1964). However, pandas surprisingly consume a large quantity of the fibrous food, and yet it is undetermind how they survive exclusively on such a diet (Schaller et al., 1985) in comparison to similarly sized herbivores. It is interesting to note that the genome of the giant panda has been reported to encode all crucial enzymes for a carnivorous digestive system but lacks those required for digesting cellulose-rich bamboo diet (Li et al., 2010). Apparently, under such circumstances, giant pandas had no other option except to be dependent on gut microbiota to adapt to a highly fibrous bamboo diet (Li et al., 2010; Xue et al., 2015). Such a dietary switch that includes a seasonal foraging shift can result in changes to the panda's GIT microbiota, leading to health problems for the host, such as inflammation to name one (Williams et al., 2016). A chronic GIT distress has been observed in captive giant pandas that results in the mucous-like stool (mucoid) (Edwards et al., 2006; Loeffler et al., 2006). Previous studies have suggested that the diet may be the primary cause of an increase in dietary proteins that are known to result in the higher mucoid occurrence of the mucous-like stool (mucoid) (Edwards et al., 2006; Janssen et al., 2006; Williams et al., 2016). Moreover, gastrointestinal disease is reported to be a primary cause of death in both wild and captive pandas (Qiu and Mainka, 1993; Janssen et al., 2006). Mainly, microorganisms link with lymphoid tissue in GIT to exclude pathogens and produce short chain fatty acids (SCFAs) (Johansson et al., 2011; Flint et al., 2012) that further helps in enhancing the barrier to pathogens (Brown et al., 2003; Louis and Flint, 2009). Therefore, it becomes most significant to study the gut microbiota and come to an understanding of the intricate biological mechanisms involved which greatly impact the panda's nutrition and health.

Ley et al. (2008) found that the giant panda gut microbiotas clustered closer to bears, and were distinct from those of other mammals. Later, Li et al. (2015b) conducted 16S rRNA based community structure analysis and found that despite sharing the same diet (bamboo) with the red panda, giant pandas harbor a more similar gut microbiota with black bears than with red pandas, which was consistent with the phylogenetic relationships but not the diet. Furthermore, a recent study on 45 captive giant pandas reported that the predominant bacterial genera in the captive giant panda were *Escherichia shigella* and *Streptococcus* bacteria, which occur in carnivores (Xue et al., 2015) including bears (Song et al., 2017). Xue et al. (2015) also suggested that the giant panda's gut microbiota structure reflects the opposite pattern from the hypothesis on mammalian gut microbiota adaptation to diet (Muegge et al., 2011), and its carnivore-like gut microbiota has not evolved to digest cellulose from a fiber-rich bamboo diet efficiently. This finding contrasts with the previous results based on three wild giant panda's gut microbiota by Zhu et al. (2011). Another study by Fang et al. (Fang et al., 2012) has found that the giant panda harbors the intestinal bacteria which aids in digesting the lignin from bamboo. But this study was based on low sample size and without any direct evidence regarding lignin digestion. Furthermore, it is difficult to come to any solid conclusions when the research is just based only on microbial composition without considering the biological function of such a community (Wei et al., 2015b). The best approach in understanding the giant panda's diet adaptability status is to have a research plan that will provide an in-depth knowledge of the panda's gut microbiota with a focus on its functional potential which clarify such condition.

Given this, we extensively analyzed 73 metagenomic data from mammals with different diets and found that the relative abundance of genes involved in cellulose- and hemicellulose-digestion, amino acid-degradation and -biosynthesis pathways in giant panda microbiomes echo a close resemblance with bears and carnivores. Moreover, the lowest cellulase and hemicellulase activity of giant panda feces compared to major herbivores provides direct evidence that the giant panda is not specialized to digest cellulose and hemicellulose in the bamboo diet.

## Materials and methods

### Sample collection

The fecal samples of captive-born giant pandas (*Ailuropoda melanoleuca*) (*n* = 6) and Asiatic black bears (*Ursus thibetanus*) (*n* = 4) were collected from the China Conservation and Research Center for the Giant Panda and Bifengxia Ecological Zoo (Ya'an, Sichuan Province, China), respectively. Four fecal samples of bamboo rats (*Rhizomys sinensis*) were collected from the Chengdu Ecological Bamboo Rat Farm (Chengdu, Sichuan Province, China) (Table S1). All samples were immediately frozen in liquid nitrogen prior to storage at −80°C until use. The samples of giant pandas and Asiatic black bears (*Ursus thibetanus*) were collected in January, 2015, and the samples of bamboo rats (*Rhizomys sinensis*) were collected in December, 2016. More than 90% of the diet of the captive giant pandas was composed of bamboo stems or leaves, with the remaining as fruits, vegetables, and corn/wheat concentrates.

For conducting an enzyme assay experiment, we collected a total of 65 fresh fecal samples from different animals including rabbit, goat, horse, cow, and mice from the farm of Sichuan Agricultural University (Ya'an, Sichuan Province, China); giraffe, zebra, argali sheep, sika deer, tiger, wolf, lion, and baboon were collected from the Bifengxia Ecological Zoo (Ya'an, Sichuan Province, China); and giant pandas from the China Conservation and Research Center (Ya'an, Sichuan Province, China) (Table S2). Fresh fecal samples of each species were immediately frozen in liquid nitrogen prior to storage at −80°C until use. All samples were collected in April, 2017.

### DNA extraction and sequencing

A frozen aliquot (500 mg) of each fecal sample was processed, and bacterial DNA was extracted using the MO BIO PowerFecal™ DNA Isolation Kit (MO BIO Laboratories, Carlsbad, CA, USA) by following the manufacturer's protocol. The DNA concentration (ranging from 15.2 to 75.4 ng/μl) of all samples was measured by NanoDrop (Thermo Scientific) and its quality was estimated on agarose gel electrophoresis. Only samples that meet the following criteria were used for library preparation: (1) DNA concentration is >15 ng/μl; (2) the total quantity of DNA is >6 μg; (3) DNA band that was visualized on agarose gel electrophoresis must be clear and of good quality. Finally, 1 μg DNA of each sample was pooled to yield an equimolar concentration to construct the DNA libraries (DNA was sheared to 350 bp) using the Illumina DNA Sample Preparation kit according to the manufacturer's instructions. Amplified libraries were sequenced on Illumina HiSeq 2500 instrument using paired-end 2 × 250 bp chemistry which was performed by Novogene (Beijing, China).

The metagenome dataset used in this study was deposited into the National Centre for Biotechnology Information's Sequence Read Archive (SRA; http://www.ncbi.nlm.nih.gov/sra) under accession bioproject number: PRJNA407583 (SRA number: SAMN07660490 - SAMN07660503).

### Shotgun metagenomic sequence analysis

Adaptor contamination was removed using cutadapt 1.3 (Martin, 2011) (https://pypi.python.org/pypi/cutadapt/1.3) with parameters “-o 4 -e 0.1.” The bamboo sequences were removed from the dataset by blasting clean reads with the genome of Moso Bamboo (*Phyllostachys heterocycla*) (http://202.127.18.221/bamboo/down.php) (Peng et al., 2013) using Burrows-Wheeler Aligner (BWA) (Li and Durbin, 2009) with default parameters (http://bio-bwa.sourceforge.net/). The 90% sequence similarity cutoff used in the filter step should have allowed us to remove most sequences belonging to different bamboo species. Reads of the giant pandas and the black bears that mapped to the genome of Giant Panda (http://asia.ensembl.org/Ailuropoda_melanoleuca/Info/Index) (Li et al., 2010) were also filtered. The host sequences of the bamboo rat were also removed by blasting with the genome of rat (ftp://ftp.ensembl.org/pub/release-70/fasta/rattus_norvegicus/dna/). Quality control was performed using a sliding window (5 bp bases) by Trimmomatic (Bolger et al., 2014) using the following criteria: (1) cutting once the average quality within the window falls below Q 20; (2) clean reads do not contain any N-bases; (3) trimming is applied to the 3‘end of reads, dropping those reads that were below 125 bp length; (4) only paired-end reads were retained for downstream analyses. To minimize the potential effects of differences in sequence process between data sets, we assembled our clean reads using FLASH software based on the overlapping sequences (Magoc and Salzberg, 2011) to match previously published data set (454 and MiSeq platform) (Muegge et al., 2011; Sanders et al., 2015). These datasets were downloaded from Metagenome Analysis Server (http://metagenomics.anl.gov). Also, gut metagenomics data of three wild giant pandas (Zhu et al., 2011) were included from IMG (https://img.jgi.doe.gov/cgi-bin/m/main.cgi) (Table S3). To reduce biases caused by different sequence depth, data including our dataset (*n* = 14) and 17 samples from the study of Sanders et al. (2015) were randomly sub-sampled to 140869 [the largest number of sequences of the samples from Muegge et al.] (Muegge et al., 2011) using seqtk-master (https://github.com/lh3/seqtk). MetaGeneMark (prokaryotic GeneMark.hmm version 2.8) (Noguchi et al., 2006) was used to predict ORFs from all sequences.

For all samples examined in this study, predicted amino acid sequences were searched against the Kyoto Encyclopedia of Genes and Genomes (KEGG) online database with parameters “for prokaryotes in representative set” (Moriya et al., 2007) and other parameters were chosen by default. Carbohydrate-active enzymes annotation was performed using the blast in CAZYmes Analysis Toolkit (CAT) (Park et al., 2010) (http://cricket.ornl.gov/cgi-bin/cat.cgi) choosing the sequence based annotation with an *E*-value cut-off of 10-5 and Bit Score = 60.

### Statistical analysis

Relative abundances of Non-eukaryotic KEGG Orthology (KO) gene and CAZyme family were calculated by normalizing all the KO and CAZy family of each sample to sum to 1, respectively. Observation matrix tables containing relative abundance information of KOs were used to calculate Euclidean distance based on UPGMA Algorithm, and Principal Coordinates Analysis (PCoA) plot was built using PAST v.3.1 data analysis package (Hammer et al., 2001). The relative enrichment of genes for cellulose- and hemicellulose- degradation including endocellulase (EC:3.2.1.4), beta-glucosidase (EC:3.2.1.21), beta-xylosidase (EC:3.2.1.37) and endo-1,4-beta-xylanase (EC:3.2.1.8, EC:3.2.1.136) were compared between giant pandas and other animals. To investigate the beta-diversity of fiber-degrading genes in the gut of mammals, we performed the distance analysis and PCoA ordination of genes for cellulose- and hemicellulose- degradation. Given the small and unequal sample size, we performed all the statistical tests for comparison between giant panda and other animals by using Mann Whitney test. For two sets of metabolic pathways, central pyruvate and glutamate metabolism which have previously been revealed as significant differentiations in herbivores and carnivores (Muegge et al., 2011; David et al., 2014), we manually drew these pathways based on differences in genes' enrichment. UPGMA-clustering tree of CAZyme was created using the relative abundance of CAZy families. We added jackknife supports at the nodes of UPGMA-clustering tree based on bootstrap 1,000 times to interpret the uncertainty between the taxonomic groups. We also compared the relative abundance of CAZy family members, which were known to be related to digest cellulose (GH5, GH6, GH7, GH9, GH44, GH45, and GH48) and hemicellulose (GH8, GH10, GH11, GH12, and GH26, GH28, GH53) (Pope et al., 2010; Zhu et al., 2011; Lombard et al., 2014) (http://www.cazy.org/) (see Table S4 for the function of these CAZy family), with other mammals. Besides, we performed the linear discriminant analysis effect size (LEfSe) that accounts for multiple testing by including Kruskal-Wallis test among classes, and Wilcoxon test between subclasses (https://huttenhower.sph.harvard.edu/galaxy/) (Segata et al., 2011). LEfSe analysis was used to perform significance test for CAZy family to identify genes differentially represented between different diet groups, with a *P*-value cut-off of 0.01 and a minimum effect size of 3 (Segata et al., 2011). The entire visualized figures were drawn by R 3.1.2. The test of significance based on Mann-Whitney test was performed to determine whether there was a significant difference abundance of gene between different diet groups (giant pandas, bears, carnivores, herbivores, and omnivores) by using GraphPad Prism 5 software (GraphPad Software, Inc., USA).

### Enzyme activity assay

To verify the cellulose degradation activity of gut microbiota in the giant panda, we quantitatively estimated the cellulase and xylanase activity in fecal samples from giant pandas, carnivores, omnivores, and herbivores by calculating degradation capacity [i.e., producing 1 μg reducing sugars per gram sample per minute under the assay conditions (Abou-Taleb et al., 2009; Gupta et al., 2012)]. Cellulase and xylanase are the most critical enzyme in the cellulose and hemicellulose-degradation pathway, respectively. So we decided to measure and compare their activity in the feces of giant pandas and other mammals. The cellulase and xylanase activity was determined using cellulase anthrone colorimetry (Black, 1951) and neutral xylanase [3,5-dinitrosalicylic acid method (Miller, 1959)] activity kit (Comin Biotechnology, Suzhou, China) according to the manufacturer's instructions. In this assay, we used a total of 65 fecal samples including four individual fecal samples from each species of carnivores (tiger, wolf and lion), omnivores (mice, black bear and baboon), and herbivores (rabbit, horse, goat, cow, giraffe, zebra, argali sheep, sika deer and bamboo rat) to compare their cellulase and xylanase activity with giant panda feces (*n* = 5) (Table S2). Three replicates were taken from each sample to provide more confident and reliable results. Mann Whitney test was used for significance test between giant panda and other diet groups.

## Results

### Metagenome based functional capacity assessment

A previous study has reported the compositional similarity of the gut microbiota of the giant panda with carnivores indicated by the 16S rDNA gene (Xue et al., 2015). To understand and determine whether this similarity has any functional support, we investigated the metabolic potential of the microbiome through shotgun metagenome sequencing of the giant pandas (*n* = 6), black bears (*n* = 4) and bamboo rats (*n* = 4).

**Figure 1:**
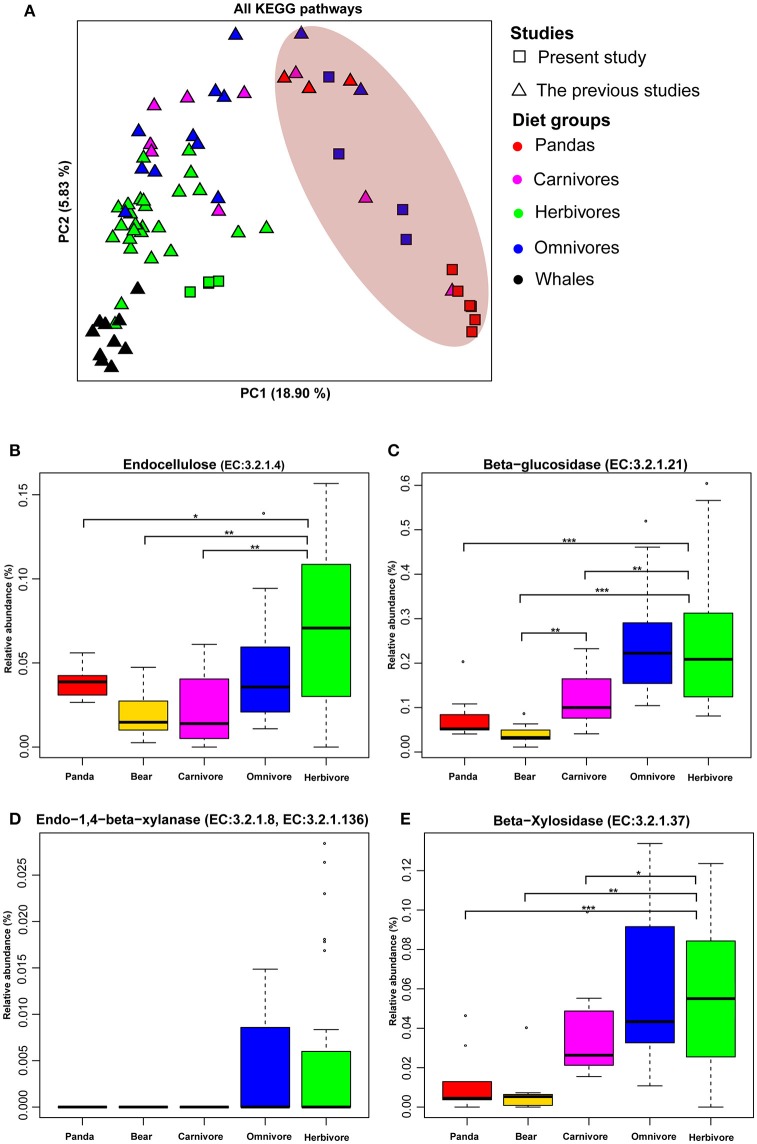
The functional compositions of giant panda microbiomes show similarity to those of bears and carnivores. **(A)** Principal components analysis ordinations of predicted metagenomic function in the gut of giant panda and terrestrial mammals when considering all pathways. Distributions of relative abundances are shown as box plots for each cellulose and hemicellulose gene: **(B)** (EC: 3.2.1.4), **(C)** (EC:3.2.1.21), **(D)** (EC:3.2.1.8, EC:3.2.1.136), and **(E)** (EC:3.2.1.37). Those gene enriched in the gut microbiota of giant panda were relatively lower than herbivores (^*^<0.05, ^**^<0.01, and ^***^<0.001 by Mann Whitney test).

A total of 207,378,961 raw paired-end reads were generated by the Hiseq 2500 platform with 2 × 250 read length. Nearly 191,853,746 high-quality paired-end reads were obtained after trimming the low-quality, host and bamboo sequences, and ultimately 169,904,552 long reads were retained by assembling for the subsequent analysis. Table S5 showed the detail of quality control and pre-processing. Additionally, a data set consisting of 74,780,464 metagenome sequences were downloaded from 59 individual samples (representing carnivores, omnivores, herbivores, whales and giant pandas) (Table S3) for combined analyses of our data. A previous study (Sanders et al., 2015) showed that whales harbor a distinct gut microbiome from those of all terrestrial mammals when considering all KEGG pathways, so we labeled it as a separate diet group in Figure 1A.

The Principle Coordinate Analysis (PCoA) of predicted metagenomic function (All KEGG pathways based on UPGMA algorithm) (Figure 1A and Figure S1) showed that the giant pandas formed a cluster with the bears (black bear, spectacled bear and polar bear) close to some carnivores and distinct from herbivores and whales. Furthermore, the Euclidean distances (Table S6) between the giant pandas and the herbivores (average value = 0.0331) was significantly larger (*P* < 0.001) than that between the giant pandas and the carnivores (average value = 0.02878) and between the giant pandas and the bears (average value = 0.02227). This result supported the similarity of the gut microbiome of giant panda and carnivores in functional capacity.

Previously, it was reported that the genes involved in cellulose (Figure S2A) and hemicellulose-degradation pathway are present in giant pandas (Zhu et al., 2011). Therefore, we examined 4 genes (endocellulase, beta-glucosidase, endo-1, 4-beta-xylanase, and beta-xylosidase), which are known to be involved in pathways of cellulose- and hemicellulose-digestion in giant pandas. We found that these four genes were present in all animal groups (including carnivores, herbivore, and omnivores) along with giant pandas (Figures S2B–E). Therefore, it is significant to evaluate the relative abundance of these genes. Similar to bears and carnivores, the relative abundances of all four genes in giant panda were significantly lower (Mann Whitney test *P* < 0.05) than those of herbivores (Figures 1B–E). In addition, giant pandas host a more similar gut microbiome to those of carnivores and bears when considering fiber-degrading enzymes (Figure S3), and the Euclidean distances of fiber-degrading enzymes (Table S7) between the giant pandas and the herbivores (average value = 0.002) was significantly larger (*P* < 0.001) than that between the giant pandas and the bears (average value = 0.00067) and that between the giant pandas and the carnivores (average value = 0.0008)

**Figure 2:**
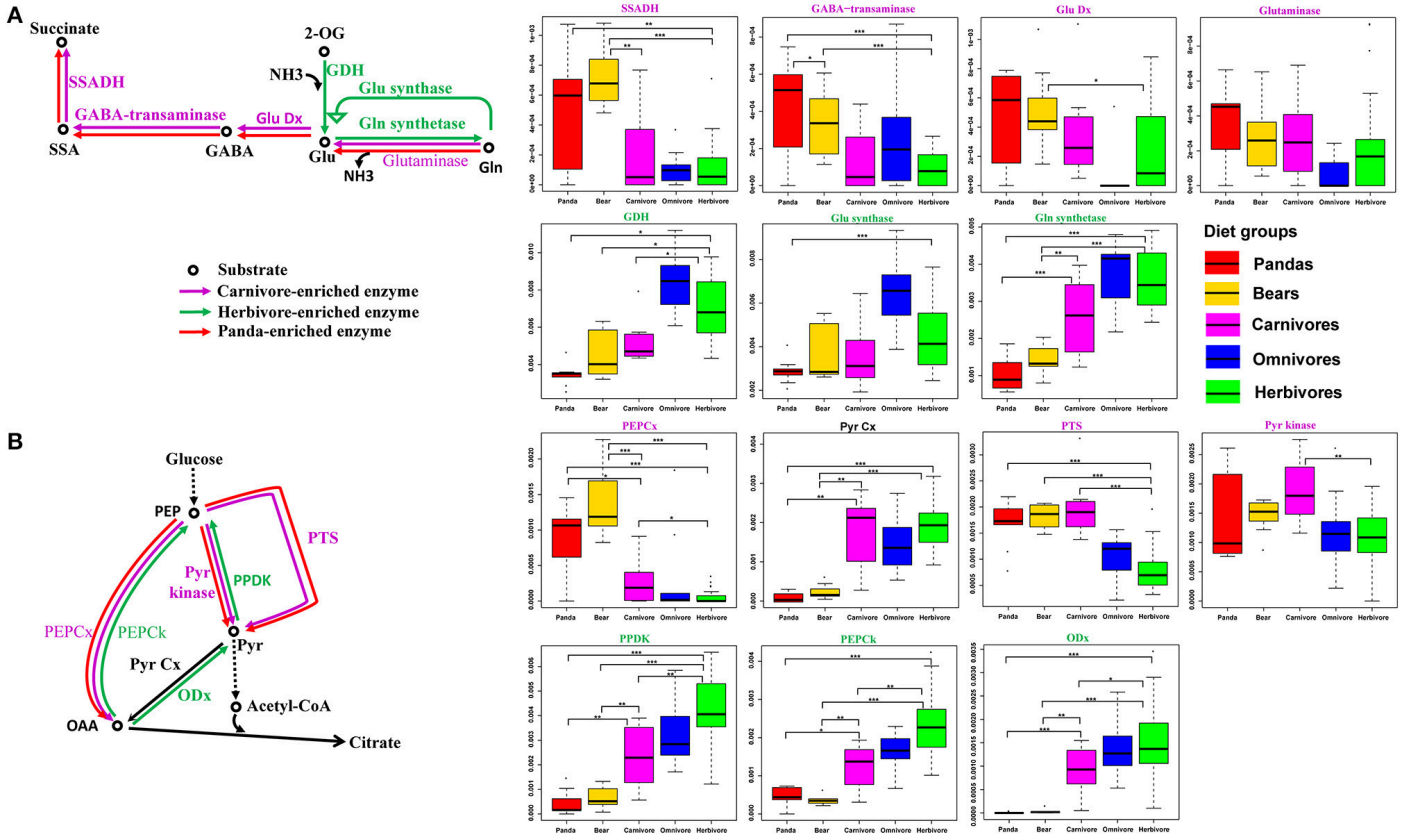
Central pyruvate- and glutamate-metabolism pathways and relative abundance of catalyzing genes in the giant pandas, bears, carnivores, omnivores, and herbivores. The giant panda gut metagenomes show a pattern of enrichment similar to carnivores in central pyruvate and glutamate metabolism. **(A)** Glutamate metabolism and distributions of relative abundances are shown as box plots for each gene in the pathways. **(B)** Pyruvate metabolism and distributions of relative abundances are shown as box plots for each gene in the pathways (^*^<0.05, ^**^<0.01, and ^***^<0.001 by Mann Whitney test).

A previous study (Muegge et al., 2011) has suggested that the herbivorous mammalian gut microbiomes are enriched in enzymes associated with amino acid biosynthetic reactions (Table S8), while gut microbiome of carnivores was enriched in enzymes related to amino acid degradation reactions. Consequently, we checked the relative abundance of enzymes involved in amino acid biosynthetic and degradation reactions. In contrast, like the carnivores, the giant panda gut microbiomes were highly enriched in enzymes associated with pathways of amino acid degradation (Figure S4), but lack the enzymes that are involved in amino acid biosynthetic reactions (Figure S5). Specifically, we examined the catabolic direction and assessed the relative abundance of genes involved in glutamate and pyruvate metabolism, which were known to be key pathways in discriminating between herbivorous and carnivorous mammals (Muegge et al., 2011; David et al., 2014), in giant pandas, bears, carnivores, omnivores and herbivores. Similar to bears and carnivores, the giant panda gut microbiota were abundant in genes involved in catalyzing the degradation of glutamine and glutamate and unlike herbivore, deficient in genes catalyzing the synthetic reactions (Figure 2A). Furthermore, in pyruvate metabolism, the giant panda gut microbiota was also abundant in genes catalyzing the degradation reactions and thus follow the similar catabolic direction as bears and carnivores (Figure 2B).

### Fermentative capability assessment

Fermentative metabolisms, including foregut and hindgut fermentation, have been considered to play essential role in utilizing the low-quality plant-based diet for herbivores (Mackie, 2002). To deduce the capacity of the intestinal fermentation in the gut of giant pandas, we compared the enzymes catalyzing the production and utilization of short chain fatty acids (SCFAs). This is based on the presumption that SCFAs are the end products of microbial fermentation (Sanders et al., 2015). Analogous to the structure of digestive tract, the giant panda and the carnivore gut microbiomes harbor a relatively low abundance of enzymes associated with the metabolism of propanoate, acetate, and butanoate (Figure S6). The abundance of the enzymes involved in the Wood-Ljungdahl and hydrogenotrophic methanogenesis pathways were also compared among herbivores, carnivores, bears and giant pandas. Not surprisingly, these enzymes were even low abundant in the gut microbiomes of giant pandas and carnivores than those of herbivores (Figure S7). Therefore, our data reveal that the short and simple GIT of the giant panda lacks the capability of microbial fermentation which is markedly similar to those of carnivores microbiomes.

### Assessment of genes encoding carbohydrate-active enzymes (CAZy)

**Figure 3:**
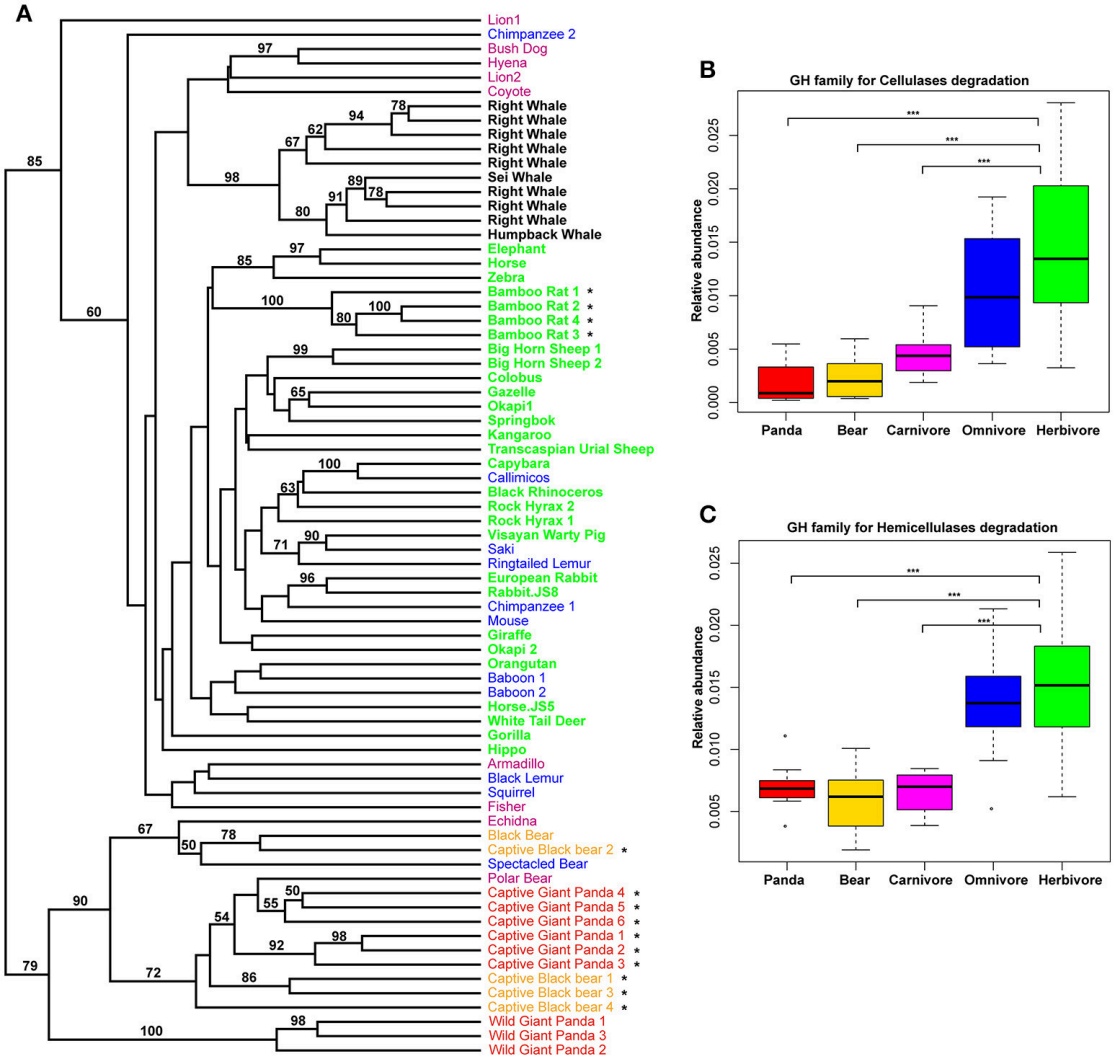
The composition of CAZymes in giant panda gut microbiomes is distinct from herbivores. UPGMA-clustering dendrogram of CAZyme relative abundances (Bootstrap values higher than 50 are shown above the node, ^*^ represent the samples which were from this study) **(A)**. The relative abundance of CAZy families for the degradation of cellulose **(B)**; and hemicellulose **(C)**. (^*^<0.05, ^**^<0.01, and ^***^<0.001 by Mann Whitney test).

Previously, it was reported that those mammals with a similar diet share the same profiles of the carbohydrate-active enzyme (CAZyme) in the gut (David et al., 2014; Sanders et al., 2015). Considering this, we examined the giant panda fecal metagenomes and successfully identified 299 different CAZy families (www.cazy.org), including 26,889 genes assigned to 93 glycoside hydrolase (GH) families. UPGMA clustering of CAZy abundance profiles including jackknife support at the nodes indicated that giant pandas grouped distinct from herbivores, but clustered together with the bears and some carnivores (Figure 3A). Among these GH families, genes that are known to have potential activity in degrading cellulose and hemicellulose (Table S4) were less abundant in giant pandas than in those of herbivores and similar to bears and carnivores (Figures 3B,C). Furthermore, we used the linear discriminant analysis effect size (LEfSe) that focuses on biological relevance along with statistical significance, to identify genes differentially represented between herbivores vs. giant panda, herbivores vs. carnivores, and carnivores vs. giant pandas. It showed that 41 and 25 CAZy families were significantly more abundant in herbivores than that in giant pandas and carnivores, respectively (Figures S8A,B). Of these, 21 families were highly abundant in herbivores while comparing to both carnivores and the giant panda. It was interesting that 5 families (GH5, GH28, GH9, GH10, GH26), involved in cellulose and hemicellulose degradation (Pope et al., 2010; Zhu et al., 2011), were significantly less abundant in the gut microbiome of giant pandas and carnivores as compared to those of herbivores (Figures S8A,B). Whereas, among the 8 CAZy families that were significantly more abundant in giant pandas than in carnivores, no CAZy family was found associated with cellulose and hemicellulose-degradation (Figure S8C). Notably, the distribution of CAZy families in the gut of giant pandas also revealed that giant pandas harbor a more similar gut function with carnivores than those of herbivores.

### Enzyme activity between giant pandas and herbivores

**Figure 4:**
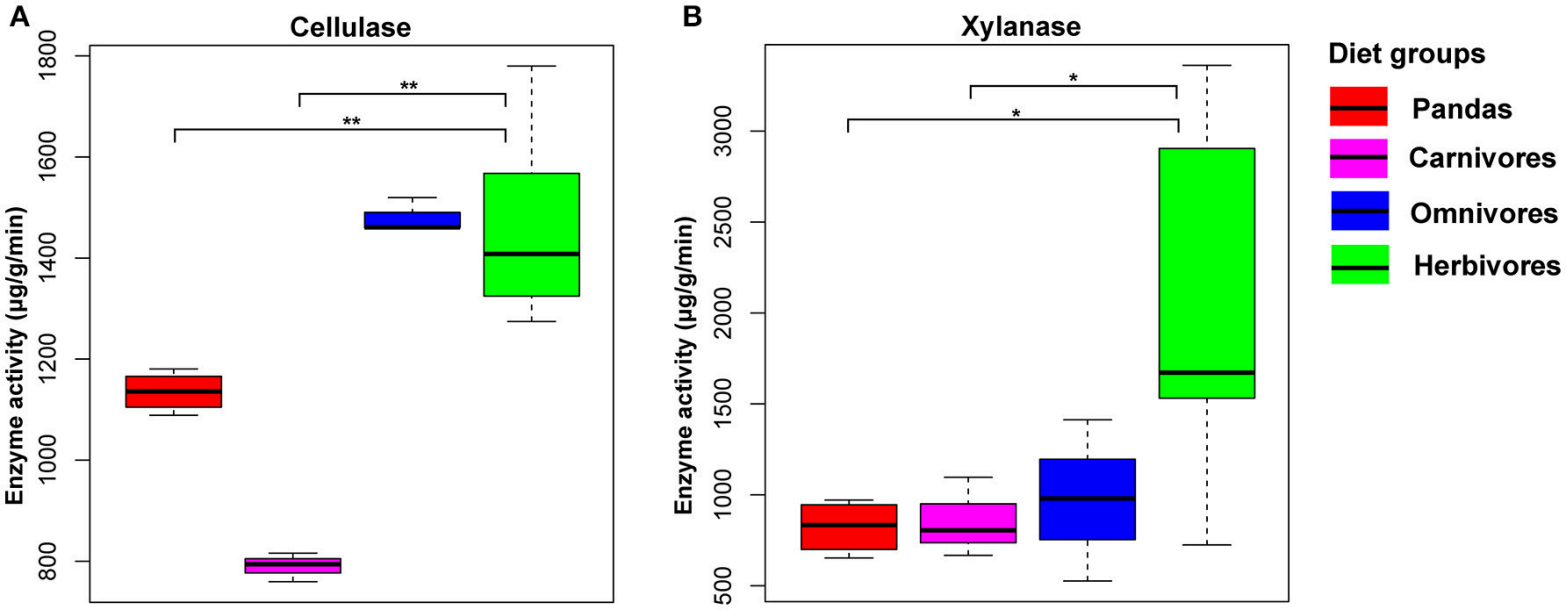
The cellulase **(A)** and hemicellulase **(B)** activity based on the mammalian feces. (significant lower, ^*^<0.05, ^**^<0.01 by Mann Whitney test).

All the above mentioned metagenomics-based results indicated that the potential capability of giant pandas in digesting cellulose and hemicellulose is not as efficient as of herbivores. To further validate this finding, it was necessary to present the direct and solid evidence of cellulose- and hemicellulose-digestion by gut microbes of giant pandas (Wei et al., 2015b). Thus, we evaluated and compared the enzyme activity for cellulose and hemicellulose-degradation among the giant pandas, herbivores, carnivores and omnivores. We observed a significant low level of cellulase (Mann Whitney test, *p* < 0.05, Figure 4A) and xylanase (Figure 4B) activity in giant panda feces than those in herbivores. In contrast, the activities of both enzymes in giant pandas were not significantly different from that of the carnivores. Furthermore, we detected that the giant panda and omnivorous bears exhibited an intermediate level of cellulose enzyme activity between carnivores and herbivores. This result provides direct evidence supporting that giant pandas lack the capability of digesting cellulose and hemicellulose from the bamboo diet.

## Discussion

Our work focuses on the gut microbiome of giant pandas that evolved from the bear family which still retain a typical carnivore-like short GIT but surprisingly feed exclusively on bamboo (Dierenfeld et al., 1982; Schaller et al., 1985; Wei et al., 2012). A previous study based on gut microbiota composition indicated by 16S rRNA gene has shown that the giant panda harbors a carnivore-like gut microbiota and may not have adapted to their bamboo diet (Xue et al., 2015). In comparison, few biologists argued that it is hard to conclude anything if the research is based on just microbial composition without considering the biological function of such a community (Wei et al., 2015b). Therefore, we investigated the gut microbiota of giant pandas with core interest to provide solid evidence of the functional capacity for the degradation of cellulose and hemicellulose.

KEGG pathways (Figure 1A and Figure S1) and CAZyme profiles (Figure 3A) indicated that the gut microbiome of the giant panda formed a cluster with bears and close to carnivores and other omnivores while distinct from herbivores and whales. Moreover, we found that the enzymes that play a significant role in degrading cellulose (Figures 1B,C, 3B) and hemicellulose (Figures 1D,E, 3C) were significantly higher in herbivores than in giant pandas, bears and carnivores. The low abundance of cellulose and hemicellulose degradation CAZymes is consistent with previous research findings (Table S9). Based on the enzyme assay, we concluded that the giant panda has the lowest cellulase (Figure 4A) and hemicellulase (Figure 4B) activity compared to major herbivores, which also compliments the results of our metagenomic analyses. This finding again supports that the giant panda's gut physiology does not aid the efficient digestion of a cellulose-rich bamboo diet. The distance analysis (Table S7) and PCoA ordination (Figure S3) of fiber-degrading enzymes showed that giant pandas harbor a more similar gut microbiome to those of bears and carnivores when considering fiber-degrading genes, which further supports our hypothesis.

Gut morphology and host phylogeny have been reported to play crucial role in shaping the gut microbiotas of animals (Ley et al., 2008; Fang et al., 2012). Phylogenetically, the giant panda belongs to family *Ursidae* (Arnason et al., 2007; Krause et al., 2008) which includes both carnivorous and omnivorous members possessing a typical carnivore-like short GIT (Sage et al., 1936; Davis, 1964). The carnivore-like gut morphology might play an important role shaping the carnivore-like gut microbiota of the giant pandas. It is also critical to consider the short evolutionary time of the giant panda compared to other specialized herbivores. We assume that the gut microbiota of the giant panda is still in the evolutionary process to acquire typical herbivorous characteristics in the future to digest fibers from the bamboo diet.

Based on the characteristics of digestive fermentation, herbivores can be classified into foregut and hindgut fermenters (Godoy-Vitorino et al., 2012). As a typical foregut fermenter, the cow has a rumen (Stewart et al., 1987) while the horse has an alternative cecum for hindgut fermentation (Hume, 1997). Whereas, giant pandas possess a typical carnivore like GIT with a straight, short and non-complex colon (10 cm in length) (Sage et al., 1936; Davis, 1964), having neither rumen nor cecum (Davis, 1964). Consequently, this may severely restrict panda's capability of fermentation. Moreover, the transit times (passage times) of bamboo in the digestive system of giant panda is reported to be short (on an average 8–10 h) (Dierenfeld et al., 1982; Schaller et al., 1985). It was remarkably faster than those of common herbivores, e.g., Wapiti (about 51 h) (Jiang and Hudson, 1996) and rabbit (approximately 61 h) (Min et al., 2013). Since the transit time of the food has been reported as the most critical factor to regulate the efficiency of consumed food utilization (Kotb and Luckey, 1972), considering such a rapid transit time and gut structure of giant panda, it is not surprising that it has low microbial fermentation efficiency for bamboo diet. Furthermore, the GIT of the giant panda does not seem to support large quantities of cellulolytic bacteria (Xue et al., 2015). In addition, our findings also highlight that the short and simple intestinal tract of the giant panda (Sage et al., 1936; Davis, 1964) may not facilitate fermentation of more complex polysaccharides from the plant-based diet (Figures S6, S7). Moreover, our results provide solid evidence to establish the fact that the efficiency of the panda's gut microbiota to digest cellulose and obtain energy from the bamboo diet is very poor. We also emphasize the need for such study in future with the focus on large sample size from different captive and wild populations.

Despite all this, the giant pandas have survived on a full-time bamboo diet for more than 2 million years (Jin et al., 2007) which raises a question, “How pandas survived on the bamboo diet?” The results of ecological, morphological and genetic studies on giant pandas provided the evidence that they are well adapted to their specialized bamboo diet (Wei et al., 2015a). For example, (1) all day eating habit of giant pandas can facilitate them to overcome the disadvantage of low-digestion efficiency for bamboo diet (10-18 kg of bamboo stems or leaves (Schaller et al., 1985); (2) giant pandas have evolved optimal feeding strategies to maximize nutritional intake [it prefers leaves, bamboo shoots, and young stems containing a higher proportion of protein and lower cellulose and lignin (Wei et al., 1999), also chooses the most nutritious bamboo species in their habitat, and has dietary shifts according to different seasons to balance needed mineral nutrients (Wei et al., 1999; Zhang et al., 2014; Nie et al., 2015a)]; (3) exceptionally low daily energy expenditure due to short distance movement per day (often between 300 and 500 m) (Schaller et al., 1985; Nie et al., 2015a), longer daily resting time (spending 41% of the daily time in resting (Schaller et al., 1985); (4) deep pelage to prevent loss of energy (significantly lower surface temperatures than other animals), reduced sizes of vital organs to minimize energy expenditure and unique mutation in the DUOX2 gene to keep low thyroid hormone levels (Nie et al., 2015b) also likely enable pandas to survive on a low intake of nutrition. Above mentioned evidence scientifically explains how the giant panda could survive on a full-time bamboo diet while it lacks the capacity of fiber fermentation.

Of note, we sequenced six gut microbiomes from captive giant pandas and combined our data with previously published gut microbiome data from wild giant pandas. Given the different feeding ecology of wild and captive giant pandas, the gut microbiotas of captive giant pandas were distinct from the wild ones (Figures 1A, 3A). However, both wild and captive giant pandas formed a cluster with bears and few carnivores, but distinct from herbivores (Figures 1A, 3A). In addition, the bioinformatics and statistical analysis were based on the combined gut microbiome data from the captive and wild giant pandas, suggesting that our data is generalizable to both captive and wild giant pandas. Consistently, Zhu et al. (2011) found that the abundance of cellulases and endo-hemicellulases in the gut of wild giant pandas was the lowest in comparison to herbivores (Table S9), this finding also agrees with the findings of the captive giant pandas in our study. Other confounding factors of our study include the different sequence platforms and studies. To assess how such confounders affected our data analysis we performed PERMONOVA analysis and found that, although different platforms (*F* = 5.7, *P* = 0.001) and studies (*F* = 4.3, *P* = 0.001) have significant effect on the metabolic potential discovered in this study, different diet groups (*F* = 9.9, *P* = 0.001) are still the largest driver of the clustering patterns (Table S10).

Our gut metagenomic findings in the giant pandas have implications in future conservation efforts. Previous ecological studies have reported the reduction in the panda's distribution, and fragmentation of their population due to the climate change, habitat loss and fragmentation during past two centuries (Hu, 2001; Li and Shen, 2012). In this study, we found that the giant panda's gut microbiota is not capable of efficiently digesting a cellulose-rich bamboo diet. This indicates that the less bamboo abundance in a fragmented or degraded forest, would affect the panda's survival since they need a greater amount of bamboo in their diet to compensate for their nutritional requirements (Schaller et al., 1985). Low digestibility and low nutrition bamboo characteristic of a degraded and fragmented forest can put the panda's survival in jeopardy. Therefore, in addition to ongoing panda conservation efforts, there is a strong need to focus on conservation of the native bamboo forests both in high- and low-altitude ranges. We propose that all these factors will be used to further strengthen conservation plans leading to the robust survival of the giant panda. Furthermore, some of the captive giant pandas will be released into the wild for conservation purposes (https://www.pandasinternational.org/). In view of this, our findings highlight the need to establish a standard for the selection of qualified captive giant pandas based on their gut microbiome in addition to other criteria practiced generally. Also, our study sets up a platform to use the giant panda as a model for future studies to investigate host-microbial interactions in animal species that show a similarly unusual and unexplored link between microbiome and diet. Such studies may provide a better understanding of how such hosts meet energy requirements and compensate for the microbiome functions and why the microbiome has or has not evolved for a particular diet.

## Availability of data and materials

The metagenome dataset used in this study was deposited into the National Centre for Biotechnology Information's Sequence Read Archive (SRA; http://www.ncbi.nlm.nih.gov/sra) under accession bioproject number: PRJNA407583.

## Ethics statement

The entire animal work was carried out under the approval of the Institutional Animal Care and Use Committee of the Sichuan Agricultural University under the permit number DKY-B20130302. All experiments were performed in accordance with the approved guidelines and regulations.

## Author contributions

Study and experiments were conceived and designed by YiL and JZ. Reagents, materials, analysis tools were contributed by BZ, RN, ML, HenZ, YuZ, YT, YiZ, HL, YuL, JY, YaL, QN, ZZ, MY, MZ, CL, CW, DL, HemZ. Data collection and experimental procedures were conducted by WG. Data analysis and interpretation were performed by WG, SM, and YuL. The manuscript was written and prepared by SM, WG, JZ, and YiL. All authors read and approved the final manuscript.

### Conflict of interest statement

The authors declare that the research was conducted in the absence of any commercial or financial relationships that could be construed as a potential conflict of interest.

## Supplementary material

Table S1 Detailed information of the fecal samples used in this study.

Table S2 Information of the fecal samples used to measure the cellulase activity.

Table S3 Information and basic statistics on the additional mammalian samples included in our comparative study.

Table S4 The function of putative cellulose- and hemicellulose-GH families.

Table S5 Summary of whole-genome shotgun (WGS) sequencing.

Table S6 Analysis of similarity of the Euclidean distances.

Table S7 Analysis of similarity of the Euclidean distances of fiber-degrading genes.

Table S8 Summary of the differences in amino acid metabolism between herbivore and carnivore microbiomes from the analysis of Muegge et al. (2011).

Table S9 Relative abundance of CAZy families involved in cellulose- and hemicellulose-degradation as reported in previous studies.

Table S10 PERMANOVA pseudo-F and *p*-values associated with specific factor.

Figure S1 Principal components analysis ordinations of predicted metagenomic function in the gut of giant panda and terrestrial mammals when considering all pathways.

Figure S2 The pathways of cellulose degradation in the gut microbiota of different mammalian dietary categories: **(A)** Schematic of pathways of cellulose digestion. **(B)** Carnivores, **(C)** Herbivores, **(D)** Omnivores, and **(E)** Giant panda. All mammalian groups harbor the common pathways for cellulose- and hemicellulose-degradation.

Figure S3 Principal components analysis ordinations of metagenomic function in the gut of giant panda and terrestrial mammals when considering fiber-degrading genes.

Figure S4 Relative abundance of enzymes involved in amino acid degradation pathways in giant pandas, bears, carnivores, ominivores, and herbivores. Except EC: 3.5.3.6 and EC: 1.5.1.12, the giant pandas reflect the similar result to those of bears and carnivores: most of the enzymes involved in amino acid-degradation pathways were more enriched than herbivores (^*^ < 0.05, ^**^ < 0.01, and ^***^ < 0.001 by Mann-Whitney test).

Figure S5 Relative abundance of enzymes involved in amino acid biosynthesis reactions in giant pandas, bears, carnivores, ominivores, and herbivores. The herbivores harbor more proportional abundance of enzymes involved in amino acid biosynthesis than giant pandas, bears, and carnivores (^*^ < 0.05, ^**^ < 0.01, and ^***^ < 0.001 by Mann-Whitney test).

Figure S6 Relative abundance of SCFA-associated enzymes in giant pandas, bears, carnivores, ominivores, and herbivores from the analysis of Sanders et al. (our data reveal the completely consistent results with Sanders et al. (2015) (^*^ < 0.05, ^**^ < 0.01, and ^***^ < 0.001 by Mann-Whitney test).

Figure S7 Relative abundance of key enzymes associated with the Wood-Ljungdahl and hydrogenotrophic methanogenesis pathway in giant pandas, bears, carnivores, ominivores, and herbivores in the analysis of Sanders et al. (2015) (our data reveals completely consistent results with Sanders et al., 2015) (^*^ < 0.05, ^**^ < 0.01, and ^***^ < 0.001 by Mann-Whitney test).

Figure S8 CAZYme families significantly differentiated among giant pandas, herbivores, and carnivores by LEfSe. (*P*-value cut-off of 0.01 and a minimum effect size of 3, ^*^ represent the GH family is correlate with cellulose and hemicellulose degradation).
