# Supplementary material for: Metagenomic Study Suggests That the Gut Microbiota of the Giant Panda (Ailuropoda melanoleuca) May Not Be Specialized for Fiber Fermentation
Source: Front Microbiol. 2018 Feb 16;9:229. doi: 10.3389/fmicb.2018.00229 (PMC5820910; doi:10.3389/fmicb.2018.00229)
Supplement: Table S1 — Detailed information of the fecal samples used in this study. [file Table1.PDF]

Table S1. Detailed information of the fecal samples used in this study.

Sample ID	Host scientific name	Host major clade	Host common name	Host family	Diet	Collection date	Location	Country	Coordinates
FY1.27	*Ailuropoda melanoleuca*	Carnivora	Giant panda	Ursidae	Bamboo	Jan 2015	China Conservation and Research Center for the Giant Panda	China	30.37753, 102.8983
DL1	*Ailuropoda melanoleuca*	Carnivora	Giant panda	Ursidae	Bamboo	Jan 2015	China Conservation and Research Center for the Giant Panda	China	30.37752, 102.8929
WG	*Ailuropoda melanoleuca*	Carnivora	Giant panda	Ursidae	Bamboo	Jan 2015	China Conservation and Research Center for the Giant Panda	China	30.33619, 102.8725
GZ1.9.2	*Ailuropoda melanoleuca*	Carnivora	Giant panda	Ursidae	Bamboo	Jan 2015	China Conservation and Research Center for the Giant Panda	China	30.37633, 102.8778
ZM2.1.27	*Ailuropoda melanoleuca*	Carnivora	Giant panda	Ursidae	Bamboo	Jan 2015	China Conservation and Research Center for the Giant Panda	China	30.38123, 102.9082
HH	*Ailuropoda melanoleuca*	Carnivora	Giant panda	Ursidae	Bamboo	Jan 2015	China Conservation and Research Center for the Giant Panda	China	30.61856, 102.8905
CB1	*Ursus thibetanus*	Carnivora	Black bear	Ursidae	Omnivorous	Jan 2015	Bifengxia Ecological Zoo	China	30.0760, 102.9895
CB3	*Ursus thibetanus*	Carnivora	Black bear	Ursidae	Omnivorous	Jan 2015	Bifengxia Ecological Zoo	China	30.0760, 102.9895
CB7	*Ursus thibetanus*	Carnivora	Black bear	Ursidae	Omnivorous	Jan 2015	Bifengxia Ecological Zoo	China	30.0760, 102.9895
CB9	*Ursus thibetanus*	Carnivora	Black bear	Ursidae	Omnivorous	Jan 2015	Bifengxia Ecological Zoo	China	30.0760, 102.9895
BR1	*Rhizomyidae*	Rodentia	Bamboo rat	Spalacidae	Herbivore	Dec 2016	Chengdu ecological Bamboo rat farm	China	30.9940,103.9318
BR2	*Rhizomyidae*	Rodentia	Bamboo rat	Spalacidae	Herbivore	Dec 2016	Chengdu ecological Bamboo rat farm	China	30.9940,103.9318
BR3	*Rhizomyidae*	Rodentia	Bamboo rat	Spalacidae	Herbivore	Dec 2016	Chengdu ecological Bamboo rat farm	China	30.9940,103.9318
BR4	*Rhizomyidae*	Rodentia	Bamboo rat	Spalacidae	Herbivore	Dec 2016	Chengdu ecological Bamboo rat farm	China	30.9940,103.9318
