# Supplementary material for: Metagenomic Study Suggests That the Gut Microbiota of the Giant Panda (Ailuropoda melanoleuca) May Not Be Specialized for Fiber Fermentation
Source: Front Microbiol. 2018 Feb 16;9:229. doi: 10.3389/fmicb.2018.00229 (PMC5820910; doi:10.3389/fmicb.2018.00229)
Supplement: Table S2 — Information of the fecal samples used to measure the cellulase activity. [file Table2.PDF]

Table S2. Information of the fecal samples used to measure the cellulose activity.

Sample number	Sample ID	Host scientific name	Host common name	Host family	Diet	Collection date	Country	Coordinates	Location
1	P1	*Ailuropoda melanoleuca*	Giant panda	Ursidae	Bamboo	Apr 2017	China	30.06285, 102.9923	China Conservation and Research Center for the giant panda
2	P2	*Ailuropoda melanoleuca*	Giant panda	Ursidae	Bamboo	Apr 2017	China	30.06285, 102.9923	China Conservation and Research Center for the giant panda
3	P3	*Ailuropoda melanoleuca*	Giant panda	Ursidae	Bamboo	Apr 2017	China	30.38123, 102.9082	China Conservation and Research Center for the giant panda
4	P4	*Ailuropoda melanoleuca*	Giant panda	Ursidae	Bamboo	Apr 2017	China	30.06285, 102.9923	China Conservation and Research Center for the giant panda
5	P5	*Ailuropoda melanoleuca*	Giant panda	Ursidae	Bamboo	Apr 2017	China	30.06285, 102.9923	China Conservation and Research Center for the giant panda
6	T1	*Oryctolagus cuniculus*	Rabbit	Leporidae	Herbivorous	Apr 2017	China	29.9814160871,102.9989882502	Farm of Sichuan Agricultural University
7	T2	*Oryctolagus cuniculus*	Rabbit	Leporidae	Herbivorous	Apr 2017	China	29.9814160871,102.9989882502	Farm of Sichuan Agricultural University
8	T3	*Oryctolagus cuniculus*	Rabbit	Leporidae	Herbivorous	Apr 2017	China	29.9814160871,102.9989882502	Farm of Sichuan Agricultural University
9	T4	*Oryctolagus cuniculus*	Rabbit	Leporidae	Herbivorous	Apr 2017	China	29.9814160871,102.9989882502	Farm of Sichuan Agricultural University
10	N1	*Bovine*	Cow	Bovidae	Herbivorous	Apr 2017	China	29.9814160871,102.9989882502	Farm of Sichuan Agricultural University
11	N2	*Bovine*	Cow	Bovidae	Herbivorous	Apr 2017	China	29.9814160871,102.9989882502	Farm of Sichuan Agricultural University
12	N3	*Bovine*	Cow	Bovidae	Herbivorous	Apr 2017	China	29.9814160871,102.9989882502	Farm of Sichuan Agricultural University
13	N4	*Bovine*	Cow	Bovidae	Herbivorous	Apr 2017	China	29.9814160871,102.9989882502	Farm of Sichuan Agricultural University
14	Y1	*Capra aegagrus hircus*	Goat	Bovidae	Herbivorous	Apr 2017	China	29.9814160871,102.9989882502	Farm of Sichuan Agricultural University
15	Y2	*Capra aegagrus hircus*	Goat	Bovidae	Herbivorous	Apr 2017	China	29.9814160871,102.9989882502	Farm of Sichuan Agricultural University
16	Y3	*Capra aegagrus hircus*	Goat	Bovidae	Herbivorous	Apr 2017	China	29.9814160871,102.9989882502	Farm of Sichuan Agricultural University

17	Y4	*Capra aegagrus hircus*	Goat	Bovidae	Herbivorous	Apr 2017	China	29.981416087 1,102.9989882 502	Farm of Sichuan Agricultural University
18	M1	*Equus ferus caballus*	Horse	Equidae	Herbivorous	Apr 2017	China	29.981416087 1,102.9989882 502	Farm of Sichuan Agricultural University
19	M2	*Equus ferus caballus*	Horse	Equidae	Herbivorous	Apr 2017	China	29.981416087 1,102.9989882 502	Farm of Sichuan Agricultural University
20	M3	*Equus ferus caballus*	Horse	Equidae	Herbivorous	Apr 2017	China	29.981416087 1,102.9989882 502	Farm of Sichuan Agricultural University
21	M4	*Equus ferus caballus*	Horse	Equidae	Herbivorous	Apr 2017	China	29.981416087 1,102.9989882 502	Farm of Sichuan Agricultural University
22	Mi1	*Mus musculus*	Mouse	Muridae	Omnivorous	Apr 2017	China	29.981416087 1,102.9989882 502	Farm of Sichuan Agricultural University
23	Mi2	*Mus musculus*	Mouse	Muridae	Omnivorous	Apr 2017	China	29.981416087 1,102.9989882 502	Farm of Sichuan Agricultural University
24	Mi3	*Mus musculus*	Mouse	Muridae	Omnivorous	Apr 2017	China	29.981416087 1,102.9989882 502	Farm of Sichuan Agricultural University
25	Mi4	*Mus musculus*	Mouse	Muridae	Omnivorous	Apr 2017	China	29.981416087 1,102.9989882 502	Farm of Sichuan Agricultural University
26	F1	*Papio hamadryas*	Baboon	Cercopith ecidae	Omnivorous	Apr 2017	China	30.06285, 102.9923	Bifengxia Ecological Zoo
27	F2	*Papio hamadryas*	Baboon	Cercopith ecidae	Omnivorous	Apr 2017	China	30.06285, 102.9923	Bifengxia Ecological Zoo
28	F3	*Papio hamadryas*	Baboon	Cercopith ecidae	Omnivorous	Apr 2017	China	30.06285, 102.9923	Bifengxia Ecological Zoo
29	F4	*Papio hamadryas*	Baboon	Cercopith ecidae	Omnivorous	Apr 2017	China	30.06285, 102.9923	Bifengxia Ecological Zoo
30	CJL1	*Giraffa camelopardali s*	Giraffe	Giraffidae	Herbivorous	Apr 2017	China	30.06285, 102.9923	Bifengxia Ecological Zoo
31	CJL2	*Giraffa camelopardali s*	Giraffe	Giraffidae	Herbivorous	Apr 2017	China	30.06285, 102.9923	Bifengxia Ecological Zoo
32	CJL3	*Giraffa camelopardali s*	Giraffe	Giraffidae	Herbivorous	Apr 2017	China	30.06285, 102.9923	Bifengxia Ecological Zoo
33	CJL4	*Giraffa camelopardali s*	Giraffe	Giraffidae	Herbivorous	Apr 2017	China	30.06285, 102.9923	Bifengxia Ecological Zoo
34	BM1	*Equus grevyi*	grevy's zebra	Equidae	Herbivorous	Apr 2017	China	30.06285, 102.9923	Bifengxia Ecological Zoo
35	BM2	*Equus grevyi*	grevy's zebra	Equidae	Herbivorous	Apr 2017	China	30.06285, 102.9923	Bifengxia Ecological Zoo

36	BM3	*Equus grevyi*	grevy's zebra	Equidae	Herbivorous	Apr 2017	China	30.06285, 102.9923	Bifengxia Ecological Zoo
37	BM4	*Equus grevyi*	grevy's zebra	Equidae	Herbivorous	Apr 2017	China	30.06285, 102.9923	Bifengxia Ecological Zoo
38	PY1	*Ovis ammon*	Argali sheep	Bovidae	Herbivorous	Apr 2017	China	30.06285, 102.9923	Bifengxia Ecological Zoo
39	PY2	*Ovis ammon*	Argali sheep	Bovidae	Herbivorous	Apr 2017	China	30.06285, 102.9923	Bifengxia Ecological Zoo
40	PY3	*Ovis ammon*	Argali sheep	Bovidae	Herbivorous	Apr 2017	China	30.06285, 102.9923	Bifengxia Ecological Zoo
41	PY4	*Ovis ammon*	Argali sheep	Bovidae	Herbivorous	Apr 2017	China	30.06285, 102.9923	Bifengxia Ecological Zoo
42	M1	*Cervus nippon*	Sika Deer	Cervidae	Herbivorous	Apr 2017	China	30.06285, 102.9923	Bifengxia Ecological Zoo
43	M2	*Cervus nippon*	Sika Deer	Cervidae	Herbivorous	Apr 2017	China	30.06285, 102.9923	Bifengxia Ecological Zoo
44	M3	*Cervus nippon*	Sika Deer	Cervidae	Herbivorous	Apr 2017	China	30.06285, 102.9923	Bifengxia Ecological Zoo
45	M4	*Cervus nippon*	Sika Deer	Cervidae	Herbivorous	Apr 2017	China	30.06285, 102.9923	Bifengxia Ecological Zoo
46	H1	*Panthera tigris*	Tiger	Felidae	Carnivorous	Apr 2017	China	30.06285, 102.9923	Bifengxia Ecological Zoo
47	H2	*Panthera tigris*	Tiger	Felidae	Carnivorous	Apr 2017	China	30.06285, 102.9923	Bifengxia Ecological Zoo
48	H3	*Panthera tigris*	Tiger	Felidae	Carnivorous	Apr 2017	China	30.06285, 102.9923	Bifengxia Ecological Zoo
49	H4	*Panthera tigris*	Tiger	Felidae	Carnivorous	Apr 2017	China	30.06285, 102.9923	Bifengxia Ecological Zoo
50	L1	*Canis lupus*	Wolf	Canidae	Carnivorous	Apr 2017	China	30.06285, 102.9923	Bifengxia Ecological Zoo
51	L2	*Canis lupus*	Wolf	Canidae	Carnivorous	Apr 2017	China	30.06285, 102.9923	Bifengxia Ecological Zoo
52	L3	*Canis lupus*	Wolf	Canidae	Carnivorous	Apr 2017	China	30.06285, 102.9923	Bifengxia Ecological Zoo
53	L4	*Canis lupus*	Wolf	Canidae	Carnivorous	Apr 2017	China	30.06285, 102.9923	Bifengxia Ecological Zoo
54	S1	*Panthera leo*	Lion	Felidae	Carnivorous	Apr 2017	China	30.06285, 102.9923	Bifengxia Ecological Zoo
55	S2	*Panthera leo*	Lion	Felidae	Carnivorous	Apr 2017	China	30.06285, 102.9923	Bifengxia Ecological Zoo
56	S3	*Panthera leo*	Lion	Felidae	Carnivorous	Apr 2017	China	30.06285, 102.9923	Bifengxia Ecological Zoo
57	S4	*Panthera leo*	Lion	Felidae	Carnivorous	Apr 2017	China	30.06285, 102.9923	Bifengxia Ecological Zoo
58	B1	*Ursus thibetanus*	Black bear	Ursidae	Omnivorous	Apr 2017	China	30.0760, 102.9895	Bifengxia Ecological Zoo
59	B2	*Ursus thibetanus*	Black bear	Ursidae	Omnivorous	Apr 2017	China	30.0760, 102.9895	Bifengxia Ecological Zoo
60	B3	*Ursus thibetanus*	Black bear	Ursidae	Omnivorous	Apr 2017	China	30.0760, 102.9895	Bifengxia Ecological Zoo
61	B4	*Ursus thibetanus*	Black bear	Ursidae	Omnivorous	Apr 2017	China	30.0760, 102.9895	Bifengxia Ecological Zoo
62	BR1	*Rhizomyidae*	Bamboo rat	Spalacida e	Herbivore	Apr 2017	China	30.9940, 103.9318	Chengdu ecological Bamboo rat farm
63	BR2	*Rhizomyidae*	Bamboo rat	Spalacida e	Herbivore	Apr 2017	China	30.9940, 103.9318	Chengdu ecological Bamboo rat farm

64	BR3	*Rhizomyidae*	Bamboo rat	Spalacidae	Herbivore	Apr 2017	China	30.9940, 103.9318	Chengdu ecological Bamboo rat farm
65	BR4	*Rhizomyidae*	Bamboo rat	Spalacidae	Herbivore	Apr 2017	China	30.9940, 103.9318	Chengdu ecological Bamboo rat farm
