# Supplementary material for: Metagenomic Study Suggests That the Gut Microbiota of the Giant Panda (Ailuropoda melanoleuca) May Not Be Specialized for Fiber Fermentation
Source: Front Microbiol. 2018 Feb 16;9:229. doi: 10.3389/fmicb.2018.00229 (PMC5820910; doi:10.3389/fmicb.2018.00229)
Supplement: Table S3 — Information and basic statistics on the additional mammalian samples included in our comparative study. [file Table3.PDF]

Table S3. Information and basic statistics on the additional mammalian samples included in our comparative study.

Sample ID	Host Common Name	Host Family	Host scientific name	Diet	Wild/captive	Country	Coordinates	Illumina technology	Sequence count	Source
AfElphSD3.MG	african savanna elephant	Elephantidae	Loxodonta africana	Herbivore	Captive	United States of America	32.736585, -117.151475	454	140869	Muegge et al. (2011)
Armadillo.MG	southern three-toed armadillo	Dasypodidae	Tolypeutes matacus	Carnivore	Captive	United States of America	38.633556, -90.29137	454	28737	Muegge et al. (2011)
BaboonSTL.MG	hamadryas baboon	Cercopithecidae	Papio hamadryas	Omnivore	Captive	United States of America	38.633556, -90.29137	454	52293	Muegge et al. (2011)
BaboonW.MG	hamadryas baboon	Cercopithecidae	Papio hamadryas	Omnivore	Wild	Namibia	-117.151475, 18.49041	454	31429	Muegge et al. (2011)
BigHornSD.MG	big horn sheep	Bovidae	Ovis canadensis	Herbivore	Captive	United States of America	32.736585, -117.151475	454	45680	Muegge et al. (2011)
BigHornW3.MG	big horn sheep	Bovidae	Ovis canadensis	Herbivore	Wild	United States of America	39.55005, -105.782067	454	51875	Muegge et al. (2011)
BlackBr2.MG	american black bear	Ursidae	Ursus americanus	Omnivore	Captive	United States of America	38.633556, -90.29137	454	68884	Muegge et al. (2011)
BlackLemur.MG	Black Lemur	Lemuridae	Eulemur macaco	Omnivore	Captive	United States of America	38.633556, -90.29137	454	37116	Muegge et al. (2011)
BlackRhino1.MG	black rhinoceros	Rhinocerotidae	Diceros bicornis	Herbivore	Captive	United States of America	38.633556, -90.29137	454	94535	Muegge et al. (2011)
BushDog1.MG	Bush Dog	Canidae	Speothos venaticus	Carnivore	Captive	United States of America	38.633556, -90.29137	454	64646	Muegge et al. (2011)
Callimicos.MG	goeldi's marmoset	Callitrichidae	Callimico goeldii	Omnivore	Captive	United States of America	38.633556, -90.29137	454	51502	Muegge et al. (2011)
Capybara.MG	Capybara	Caviidae	Hydrochoerus hydrochaeris	Herbivore	Captive	United States of America	38.633556, -90.29137	454	107461	Muegge et al. (2011)
Chimp1.MG	Chimpanzees	Hominidae	Pan troglodytes	Omnivore	Captive	United States of America	38.633556, -90.29137	454	65969	Muegge et al. (2011)
Chimp2.MG	Chimpanzees	Hominidae	Pan troglodytes	Omnivore	Captive	United States of America	38.633556, -90.29137	454	19568	Muegge et al. (2011)
Colobus.MG	Colobus Monkey	Cercopithecidae	Colobus polykomos	Herbivore	Captive	United States of America	38.633556, -90.29137	454	67621	Muegge et al. (2011)
Echidna.MG	short-nosed echidna	Tachyglossidae	Tachyglossus aculeatus	Carnivore	Captive	United States of America	38.633556, -90.29137	454	65266	Muegge et al. (2011)

Gazelle3.MG	speke's gazelle	Bovidae	Gazella spekei	Herbivore	Captive	United States of America	38.633556, -90.29137	454	68262	Muegge et al. (2011)
Giraffe2.MG	Giraffe	Giraffidae	Giraffa camelopardalis	Herbivore	Captive	United States of America	38.633556, -90.29137	454	53668	Muegge et al. (2011)
GorillaSTL.MG	Gorilla	Hominidae	Gorilla gorilla	Herbivore	Captive	United States of America	38.633556, -90.29137	454	24933	Muegge et al. (2011)
Horse1.MG	Horse	Equidae	Equus ferus caballus	Herbivore	Wild	United States of America	38.633556, -90.29137	454	98800	Muegge et al. (2011)
Hyena2.MG	Hyena	Hyaenidae	Hyaenidae	Carnivore	Captive	United States of America	38.633556, -90.29137	454	68456	Muegge et al. (2011)
HyraxSD.MG	cape rock hyrax	Procaviidae	Procavia capensis	Herbivore	Wild	United States of America	38.633556, -90.29137	454	42140	Muegge et al. (2011)
HyraxSTL.MG	cape rock hyrax	Procaviidae	Procavia capensis	Herbivore	Captive	United States of America	32.736585, -117.151475	454	66102	Muegge et al. (2011)
Kroo3.MG	red kangaroo	Macropodidae	Macropus rufus	Herbivore	Captive	United States of America	38.633556, -90.29137	454	22049	Muegge et al. (2011)
Lion1.MG	Lion	Felidae	Panthera leo	Carnivore	Captive	United States of America	38.633556, -90.29137	454	14236	Muegge et al. (2011)
Lion2.MG	Lion	Felidae	Panthera leo	Carnivore	Captive	United States of America	38.633556, -90.29137	454	75012	Muegge et al. (2011)
Okapi1.MG	okapi	Giraffidae	Okapia johnstoni	Herbivore	Captive	United States of America	38.633556, -90.29137	454	32804	Muegge et al. (2011)
Okapi2.MG	okapi	Giraffidae	Okapia johnstoni	Herbivore	Captive	United States of America	38.633556, -90.29137	454	22615	Muegge et al. (2011)
Orang1.MG	sumatran orangutan	Hominidae	Pongo abellii	Herbivore	Captive	United States of America	38.633556, -90.29137	454	29219	Muegge et al. (2011)
PolarBr2.MG	Polar Bear	Ursidae	Ursus maritimus	Carnivore	Captive	United States of America	38.633556, -90.29137	454	56380	Muegge et al. (2011)
Rabbit.MG	european rabbit	Leporidae	Oryctolagus cuniculus	Herbivore	Captive	United States of America	38.633556, -90.29137	454	69106	Muegge et al. (2011)
RTLemur.MG	Lemuridae	ring tailed lemur	Lemur catta	Omnivore	Captive	United States of America	38.633556, -90.29137	454	50463	Muegge et al. (2011)
Saki.MG	Saki	Galagidae	Chiropotes albinasus	Omnivore	Captive	United States of America	38.633556, -90.29137	454	127069	Muegge et al. (2011)
SpecBr2.MG	Spectacled Bear	Ursidae	Tremarctos ornatus	Omnivore	Captive	United States of America	38.633556, -90.29137	454	38999	Muegge et al. (2011)

SpgbkW.MG	springbok	Bovidae	Antidorcas marsupialis	Herbivore	Captive	Namibia	-117.151475, 18.49041	454	48935	Muegge et al. (2011)
Squirrel.MG	Squirrel	Sciuridae	Sciurus vulgaris Linneaeus	Omnivore	Captive	United States of America	38.633556, -90.29137	454	43724	Muegge et al. (2011)
Urial2.MG	Transcaspian Urial Sheep	Bovidae	Ovis ammon severtzovi	Herbivore	Captive	United States of America	38.633556, -90.29137	454	31996	Muegge et al. (2011)
VWPig.MG	Visayan Warty Pig	Suidae	Phacochoerus africanus	Herbivore	Captive	United States of America	32.736585, -117.151475	454	59174	Muegge et al. (2011)
ZebraSTL1.MG	grevy's zebra	Equidae	Equus grevyi	Herbivore	Captive	United States of America	38.633556, -90.29137	454	25693	Muegge et al. (2011)
RightWhale.F5.MG	Right Whale	Balaenidae	Eubalaena glacialis	Carnivore	Captive	Atlantic Ocean	44.667833, -66.370333	illumina Hiseq	12926458	Sanders et al.(2016)
RightWhale.F8.MG	Right Whale	Balaenidae	Eubalaena glacialis	Carnivore	Captive	Atlantic Ocean	44.647667, -66.421567	illumina Hiseq	9125174	Sanders et al.(2016)
RightWhale.F12.MG	Right Whale	Balaenidae	Eubalaena glacialis	Carnivore	Captive	Atlantic Ocean	44.613667, -66.464833	illumina Hiseq	8683522	Sanders et al.(2016)
RightWhale.F16.MG	Right Whale	Balaenidae	Eubalaena glacialis	Carnivore	Captive	Atlantic Ocean	44, -66	illumina Hiseq	11603088	Sanders et al.(2016)
SeiWhale.JS1.MG	Sei Whale	Balaenopteridae	Balaenoptera borealis	Carnivore	Captive	Atlantic Ocean	44, -66	illumina Hiseq	10478824	Sanders et al.(2016)
Coyote.JS3.MG.2	Coyote	Canidae	Canis latrans	Carnivore	Captive	United States of America	42.5, -71.3	illumina Miseq	1505823	Sanders et al.(2016)
Fisher.JS7.MG.2	Fisher	Mustelidae	Martes pennanti	Carnivore	Captive	United States of America	42.5, -71.4	illumina Miseq	1233717	Sanders et al.(2016)
Horse.JS5.MG.2	Horse	Equidae	Equus ferus caballus	Herbivore	Captive	United States of America	42.5, -71.3	illumina Miseq	1119249	Sanders et al.(2016)
Mouse.JS4.MG.2	Mouse	Muridae	Mus musculus	Omnivore	Captive	United States of America	42.4, -71.1	illumina Miseq	1235292	Sanders et al.(2016)
Rabbit.JS8.MG.2	Rabbit	Leporidae	Oryctolagus cuniculus	Herbivore	Captive	United States of America	42.5, -71.3	illumina Miseq	1379739	Sanders et al.(2016)
RightWhale.F12.MG.2	Right Whale	Balaenidae	Eubalaena glacialis	Carnivore	Captive	Atlantic Ocean	44.613667, -66.464833	illumina Miseq	3136908	Sanders et al.(2016)
RightWhale.F16.MG.2	Right Whale	Balaenidae	Eubalaena glacialis	Carnivore	Captive	Atlantic Ocean	44, -66	illumina Miseq	2310842	Sanders et al.(2016)
WhitetailDeer.JS6.MG.2	White-tailed Deer	Cervidae	Odocoileus virginianus	Herbivore	Captive	United States of America	42.5, -71.3	illumina Miseq	1262255	Sanders et al.(2016)
RightWhale.F9.MG.2	Right Whale	Balaenidae	Eubalaena glacialis	Carnivore	Captive	Atlantic Ocean	44, -66	illumina Miseq	978625	Sanders et al.(2016)
RightWhale.F11.MG.2	Right Whale	Balaenidae	Eubalaena glacialis	Carnivore	Captive	Atlantic Ocean	44.613667, -66.464833	illumina Miseq	1105253	Sanders et al.(2016)

Humpback Whale.JS10.MG.2	Humpback Whale	Balaenopteridae	Megaptera novaeangliae	Carnivore	Captive	Pacific Ocean	56.9, -135.6	illumina Miseq	1016276	Sanders et al.(2016)
Hippo.JS19.MG.2	Hippopotamus	Hippopotamidae	Hippopotamus amphibius	Herbivore	Captive	Kenya	-1.3, 35.1	illumina Miseq	3532793	Sanders et al.(2016)
W1	Giant panda	Ursidae	Ailuropoda melanoleuca	Herbivore	Wild	China	<not provided>	illumina Miseq	8910	Zhu et al.(2011)
W2	Giant panda	Ursidae	Ailuropoda melanoleuca	Herbivore	Wild	China	<not provided>	illumina Miseq	16328	Zhu et al.(2011)
W3	Giant panda	Ursidae	Ailuropoda melanoleuca	Herbivore	Wild	China	<not provided>	illumina Miseq	26364	Zhu et al.(2011)

Muegge, B.D., Kuczynski, J., Knights, D., Clemente, J.C., Gonzalez, A., Fontana, L., Henrissat, B., Knight, R. & Gordon, J.I. (2011) Diet drives convergence in gut microbiome functions across mammalian phylogeny and within humans. Science, 332, 970-974.

Sanders, J.G., Beichman, A.C., Roman, J., Scott, J.J., Emerson, D., McCarthy, J.J. & Girguis, P.R. (2015) Baleen whales host a unique gut microbiome with similarities to both carnivores and herbivores. Nat Commun, 6, 8285.

Zhu, L.F., Wu, Q., Dai, J.Y., Zhang, S.N. & Wei, F.W. (2011) Evidence of cellulose metabolism by the giant panda gut microbiome. Proc Natl Acad Sci U S A, 108, 17714-17719.
