# Supplementary material for: Metagenomic Study Suggests That the Gut Microbiota of the Giant Panda (Ailuropoda melanoleuca) May Not Be Specialized for Fiber Fermentation
Source: Front Microbiol. 2018 Feb 16;9:229. doi: 10.3389/fmicb.2018.00229 (PMC5820910; doi:10.3389/fmicb.2018.00229)
Supplement: Table S4 — The function of putative cellulose- and hemicellulose-GH families. [file Table4.PDF]

Table S4. The function of putative cellulose- and hemicellulose-GH families.

CAZy family	Known activities*
GH5	Endo-β-1,4-glucanase / cellulose; endo-β-1,4-xylanase; β-glucosidase; others
GH6	Endoglucanase; cellobiohydrolase
GH7	Endo-β-1,4-glucanase; endo-β-1,3-1,4-glucanase; others
GH9	Endoglucanase; endo-β-1,3(4)-glucanase; β-glucosidase; others
GH44	Endoglucanase; xyloglucanase
GH45	Endoglucanase
GH48	Endo-β-1,4-glucanase; others
GH8	Cellulose; endo-1,4-β-xylanase; others
GH10	Endo-1,4-β-xylanase; endo-1,3-β-xylanase; others
GH11	Endo-β-1,4-xylanase; endo-β-1,3-xylanase
GH12	Endoglucanase; β-1,3-1,4-glucanase; xyloglucan hydrolase; others
GH26	β-1,3-xylanase; endo-β-1,3-1,4-glucanase; others
GH28	Polygalacturonase, rhamnogalacturonase, others
GH53	Endo-β-1,4-galactanase

***Known activities of CAZy family are from the CAZy family Carbohydrate Active Enzymes database (http://www.cazy.org/) (Lombard et al., 2014).**

Lombard, V., Golaconda Ramulu, H., Drula, E., Coutinho, P.M. & Henrissat, B. (2014) The carbohydrate-active enzymes database (CAZy) in 2013. *Nucleic Acids Res,* **42,** D490-495.
