# Supplementary material for: Metagenomic Study Suggests That the Gut Microbiota of the Giant Panda (Ailuropoda melanoleuca) May Not Be Specialized for Fiber Fermentation
Source: Front Microbiol. 2018 Feb 16;9:229. doi: 10.3389/fmicb.2018.00229 (PMC5820910; doi:10.3389/fmicb.2018.00229)
Supplement: Table S5 — Summary of whole-genome shotgun (WGS) sequencing. [file Table5.PDF]

**Table S5. Summary of whole-genome shotgun (WGS) sequencing.**

| Sample ID | Raw reads | Sequencing platform | Host and bamboo removed reads | Dropping the single reads | Final paired-end clean reads |
|---|---|---|---|---|---|
| HH | 11,477,287 × 2 | Illumina Hiseq2500 | 506,186 × 2 | 447,562 × 2 | 10,523,539 × 2 |
| DL1 | 11,245,869 × 2 | Illumina Hiseq2500 | 308,986 × 2 | 438,119 × 2 | 10,498,764 × 2 |
| FY1.27 | 12,404,708 × 2 | llumina Hiseq2500 | 677,037 × 2 | 493,722 × 2 | 11,233,949 × 2 |
| ZM2.1.27 | 12,611,226 × 2 | Illumina Hiseq2500 | 91,620 × 2 | 476,480 × 2 | 12,043,126 × 2 |
| WG | 12,489,755 × 2 | Illumina Hiseq2500 | 257,460 × 2 | 2,011,437 × 2 | 10,220,858 × 2 |
| GZ1.9.2 | 11,944,071 × 2 | Illumina Hiseq2500 | 208,976 × 2 | 412,976 × 2 | 11,322,119 × 2 |
| CB1 | 8678339 × 2 | Illumina Hiseq2500 | 8671658 × 2 | 243799 × 2 | 8427859 × 2 |
| CB3 | 8052349 × 2 | Illumina Hiseq2500 | 8050599 × 2 | 200113 × 2 | 7850486 × 2 |
| CB7 | 10906235 × 2 | Illumina Hiseq2500 | 10898856 × 2 | 425100 × 2 | 10473756 × 2 |
| CB9 | 20539115 × 2 | Illumina Hiseq2500 | 20296888 × 2 | 1317461 × 2 | 18979427 × 2 |
| BR1 | 20834283 × 2 | Illumina Hiseq2500 | 20831736 × 2 | 1550507 × 2 | 19281229 × 2 |
| BR2 | 23292067 × 2 | Illumina Hiseq2500 | 23289323 × 2 | 1796228 × 2 | 21493095 × 2 |
| BR3 | 22178991 × 2 | Illumina Hiseq2500 | 21685760 × 2 | 1483434 × 2 | 20202326 × 2 |
| BR4 | 20724666 × 2 | Illumina Hiseq2500 | 20720184 × 2 | 1416971 × 2 | 19303213 × 2 |
