# Supplementary material for: Metagenomic Study Suggests That the Gut Microbiota of the Giant Panda (Ailuropoda melanoleuca) May Not Be Specialized for Fiber Fermentation
Source: Front Microbiol. 2018 Feb 16;9:229. doi: 10.3389/fmicb.2018.00229 (PMC5820910; doi:10.3389/fmicb.2018.00229)
Supplement: Table S6 — Analysis of similarity of the Euclidean distances. [file Table6.PDF]

Table S6. Analysis of Similarity of the Euclidean distances.

	Carnivores	Herbivores	Bears	Giant pandas
Carnivores	0	0.04711	0.02616	0.02878
Herbivores	0.04711	0	0.02939	0.0331
Bears	0.02616	0.02939	0	0.02227
Giant pandas	0.02878	0.0331	0.02227	0
