# Supplementary material for: Metagenomic Study Suggests That the Gut Microbiota of the Giant Panda (Ailuropoda melanoleuca) May Not Be Specialized for Fiber Fermentation
Source: Front Microbiol. 2018 Feb 16;9:229. doi: 10.3389/fmicb.2018.00229 (PMC5820910; doi:10.3389/fmicb.2018.00229)
Supplement: Table S7 — Analysis of similarity of the Euclidean distances of fiber-degrading genes. [file Table7.PDF]

Table S7. Analysis of Similarity of the Euclidean distances of fiber-degrading genes.

	Carnivores	Herbivores	Bears	Giant pandas
Carnivores	0	0.0018	0.0008	0.0008
Herbivores	0.0018	0	0.0021	0.0020
Bears	0.0008	0.0021	0	0.00067
Giant pandas	0.0008	0.0020	0.00067	0
