# Supplementary material for: Metagenomic Study Suggests That the Gut Microbiota of the Giant Panda (Ailuropoda melanoleuca) May Not Be Specialized for Fiber Fermentation
Source: Front Microbiol. 2018 Feb 16;9:229. doi: 10.3389/fmicb.2018.00229 (PMC5820910; doi:10.3389/fmicb.2018.00229)
Supplement: Table S8 — Summary of the differences in amino acid metabolism between herbivore and carnivore microbiomes from the analysis of Muegge et al. (2011). [file Table8.PDF]

Table S8. Summary of the differences in amino acid metabolism between herbivore and carnivore microbiomes from the analysis of Muegge et al. (2008). # EC:1.3.99.2, EC:15.99.8 and EC:1.5.1.12 had been modified to EC:1.3.8.1 (in 2011 year), EC:1.5.5.2 (in 2013 year) and EC:1.2.1.88 (in 2008 year)

Amino Acid	Biosynthetic Reactions	Degradative Reactions
Alanine	No difference	No difference
Arginine	EC:2.3.1.1, EC:2.3.1.35(Significantly higher in Herbivores)	EC:3.5.3.6 (Significantly higher in Carnivores)
Asparagine	No difference	No difference
Aspartic Acid	No difference	EC:4.1.1.12 (Significantly higher in Carnivores)
Cysteine	EC:2.5.1.49 (Significantly higher in Herbivores)	No difference
Glutamic Acid	EC:1.4.1.4, EC:1.4.1.13, EC:1.4.1.14 (Significantly higher in Herbivores)	EC:4.1.1.15, EC:2.6.1.19, EC:1.2.1.16 (Significantly higher in Carnivores)
Glutamine	EC:6.3.1.2 (Significantly higher in Herbivores)	EC:3.5.1.2 (Significantly higher in Carnivores)
Glycine	EC:2.1.2.1 (Significantly higher in Herbivores)	No difference
Histidine	EC:1.1.1.23 (Significantly higher in Herbivores)	No difference
Isoleucine	No difference	EC:1.2.7.7, EC:1.2.4.4,EC:1.3.99.2# (EC:1.3.8.1) (Significantly higher in Herbivores)
Leucine	No difference	EC:1.2.7.7, EC:1.2.4.4 (Significantly higher in Herbivores)
Lysine	EC:1.5.1.7, EC:3.5.1.- (Significantly higher in Herbivores)	EC:4.1.1.8 (Significantly higher in Carnivores)
Methionine	EC:2.1.1.13,EC:2.1.1.37 (Significantly higher in Herbivores), EC:2.5.1.6, EC3.3.1.1 (Significantly higher in Herbivores)	No difference
Phenylalanine	EC:4.2.3.5 (Significantly higher in Herbivores)	No difference
Proline	No difference	EC:15.99.8 # (EC:1.5.5.2), EC:1.5.1.12 # (EC:1.2.1.88) (Significantly higher in Carnivores)
Serine	EC:1.1.1.95,EC:2.6.1.52 (Significantly higher in Herbivores)	EC:4.3.1.17, EC:4.3.1.19 (Significantly higher in Carnivores)
Threonine	No difference	EC:4.3.1.19 (Significantly higher in Carnivores)
Tryptophan	EC:4.2.3.5, EC:4.2.1.20 (Significantly higher in Herbivores)	EC:1.14.13.-, EC:2.5.1.- (Significantly higher in Carnivores)
Tyrosine	EC:4.2.3.5 (Significantly higher in Herbivores)	No difference
Valine	No difference	EC:1.2.77, EC:1.2.4.4, EC:1.3.99.2# (EC:1.3.8.1) (Significantly higher in Herbivores)

Muegge, B.D., Kuczynski, J., Knights, D., Clemente, J.C., Gonzalez, A., Fontana, L., Henrissat, B., Knight, R. & Gordon, J.I. (2011) Diet drives convergence in gut microbiome functions across mammalian phylogeny and within humans. *Science,* **332,** 970-974.
