# Supplementary material for: Metagenomic Study Suggests That the Gut Microbiota of the Giant Panda (Ailuropoda melanoleuca) May Not Be Specialized for Fiber Fermentation
Source: Front Microbiol. 2018 Feb 16;9:229. doi: 10.3389/fmicb.2018.00229 (PMC5820910; doi:10.3389/fmicb.2018.00229)
Supplement: Table S9 — Relative abundance of CAZy families involved in cellulose- and hemicellulose-degradation as reported in previous studies. [file Table9.PDF]

Table S9. Relative abundance of CAZy families involved in cellulose- and hemicellulose-degradation as reported in previous studies.

Species	Abundance of Cellulases and endohemicellulases family (in %)	References
Wild giant panda	2%	(Zhu *et al.* 2011)
Wallaby	7%	(Pope *et al.* 2010)
Termite	25%	(Warnecke *et al.* 2007)
Cow	6%	(Brulc *et al.* 2009)
Human	7%	(Qin *et al.* 2010)
Elephant	7%	(Ilmberger *et al.* 2014)
Buffalo rumen	6%	(Singh *et al.* 2014)

Additional References

Brulc, J.M., Antonopoulos, D.A., Miller, M.E., Wilson, M.K., Yannarell, A.C., Dinsdale, E.A., Edwards, R.E., Frank, E.D., Emerson, J.B., Wacklin, P., Coutinho, P.M., Henrissat, B., Nelson, K.E. & White, B.A. (2009) Gene-centric metagenomics of the fiber-adherent bovine rumen microbiome reveals forage specific glycoside hydrolases. *Proc Natl Acad Sci U S A,* **106,** 1948-1953.

Ilmberger, N., Gullert, S., Dannenberg, J., Rabausch, U., Torres, J., Wemheuer, B., Alawi, M., Poehlein, A., Chow, J., Turaev, D., Rattei, T., Schmeisser, C., Salomon, J., Olsen, P.B., Daniel, R., Grundhoff, A., Borchert, M.S. & Streit, W.R. (2014) A comparative metagenome survey of the fecal microbiota of a breast- and a plant-fed Asian elephant reveals an unexpectedly high diversity of glycoside hydrolase family enzymes. *Plos One,* **9,** e106707.

Pope, P.B., Denman, S.E., Jones, M., Tringe, S.G., Barry, K., Malfatti, S.A., McHardy, A.C., Cheng, J.F., Hugenholtz, P., McSweeney, C.S. & Morrison, M. (2010) Adaptation to herbivory by the Tammar wallaby includes bacterial and glycoside hydrolase profiles different from other herbivores. *Proc Natl Acad Sci U S A,* **107,** 14793-14798.

Qin, J., Li, R., Raes, J., Arumugam, M., Burgdorf, K.S., Manichanh, C., Nielsen, T., Pons, N., Levenez, F., Yamada, T., Mende, D.R., Li, J., Xu, J., Li, S., Li, D., Cao, J., Wang, B., Liang, H., Zheng, H., Xie, Y., Tap, J., Lepage, P., Bertalan, M., Batto, J.M., Hansen, T., Le Paslier, D., Linneberg, A., Nielsen, H.B., Pelletier, E., Renault, P., Sicheritz-Ponten, T., Turner, K., Zhu, H., Yu, C., Li, S., Jian, M., Zhou, Y., Li, Y., Zhang, X., Li, S., Qin, N., Yang, H., Wang, J., Brunak, S., Dore, J., Guarner, F., Kristiansen, K., Pedersen, O., Parkhill, J., Weissenbach, J., Meta, H.I.T.C., Bork, P., Ehrlich, S.D. & Wang, J. (2010) A human gut microbial gene catalogue established by metagenomic sequencing. *Nature,* **464,** 59-65.

Singh, K.M., Reddy, B., Patel, D., Patel, A.K., Parmar, N., Patel, A., Patel, J.B. & Joshi, C.G. (2014) High potential source for biomass degradation enzyme discovery and environmental aspects revealed through metagenomics of Indian buffalo rumen. *Biomed Research International,* **2014,** 267189.

Warnecke, F., Luginbuhl, P., Ivanova, N., Ghassemian, M., Richardson, T.H., Stege, J.T., Cayouette, M.,

McHardy, A.C., Djordjevic, G., Aboushadi, N., Sorek, R., Tringe, S.G., Podar, M., Martin, H.G., Kunin, V., Dalevi, D., Madejska, J., Kirton, E., Platt, D., Szeto, E., Salamov, A., Barry, K., Mikhailova, N., Kyrpides, N.C., Matson, E.G., Ottesen, E.A., Zhang, X., Hernandez, M., Murillo, C., Acosta, L.G., Rigoutsos, I., Tamayo, G., Green, B.D., Chang, C., Rubin, E.M., Mathur, E.J., Robertson, D.E., Hugenholtz, P. & Leadbetter, J.R. (2007) Metagenomic and functional analysis of hindgut microbiota of a wood-feeding higher termite. *Nature,* **450,** 560-565.

Zhu, L.F., Wu, Q., Dai, J.Y., Zhang, S.N. & Wei, F.W. (2011) Evidence of cellulose metabolism by the giant panda gut microbiome. *Proc Natl Acad Sci U S A,* **108,** 17714-17719.
