# Supplementary material for: Metagenomic Study Suggests That the Gut Microbiota of the Giant Panda (Ailuropoda melanoleuca) May Not Be Specialized for Fiber Fermentation
Source: Front Microbiol. 2018 Feb 16;9:229. doi: 10.3389/fmicb.2018.00229 (PMC5820910; doi:10.3389/fmicb.2018.00229)
Supplement: Table S10 — PERMANOVA pseudo-F and p-values associated with specific factor. [file Table10.PDF]

Table S10. PERMANOVA pseudo-F and p-values associated with specific factor.

Factors	Variables (Groups)	Bray–Curtis	
		pseudo-F	p-value
Diets	5(Carnivores/Herbivores/Omnivores/Pandas/Whales)	9.8698	0.001
Platforms	3(454/Miseq/Hiseq)	5.6701	0.001
Studies	4 (*Muegge et al.*/*Sanders et al.*/*Zhu et al.*/Present study)	4.2870	0.001

***number of permutations: 999**
