# Supplementary figures and images for: Metagenomic Study Suggests That the Gut Microbiota of the Giant Panda (Ailuropoda melanoleuca) May Not Be Specialized for Fiber Fermentation
Source: Front Microbiol. 2018 Feb 16;9:229. doi: 10.3389/fmicb.2018.00229 (PMC5820910; doi:10.3389/fmicb.2018.00229)

Figure S1

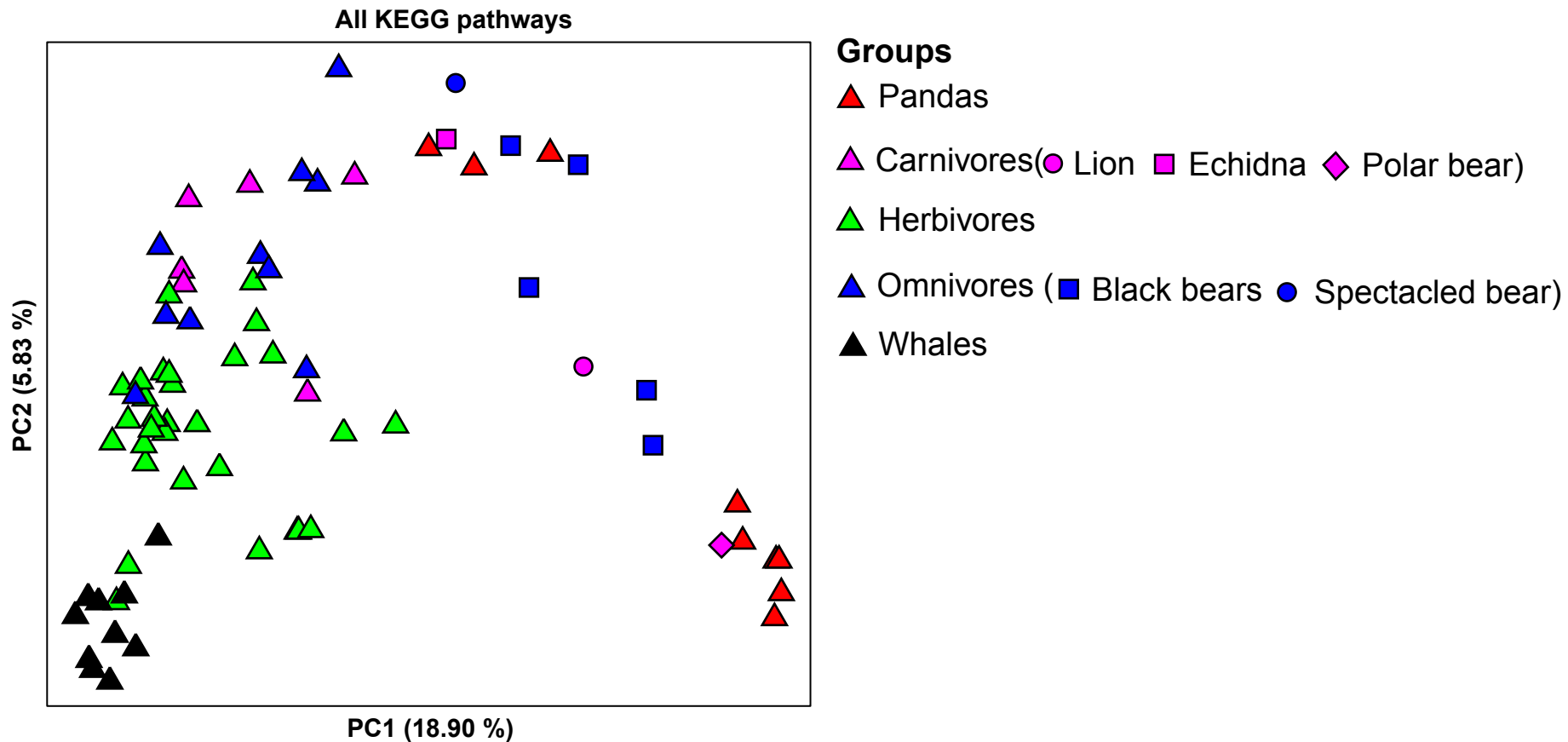

Supplement: Figure S1 — Principal components analysis ordinations of predicted metagenomic function in the gut of giant panda and terrestrial mammals when considering all pathways. [file Image1.PDF]

## B Carnivores

## B Carnivores

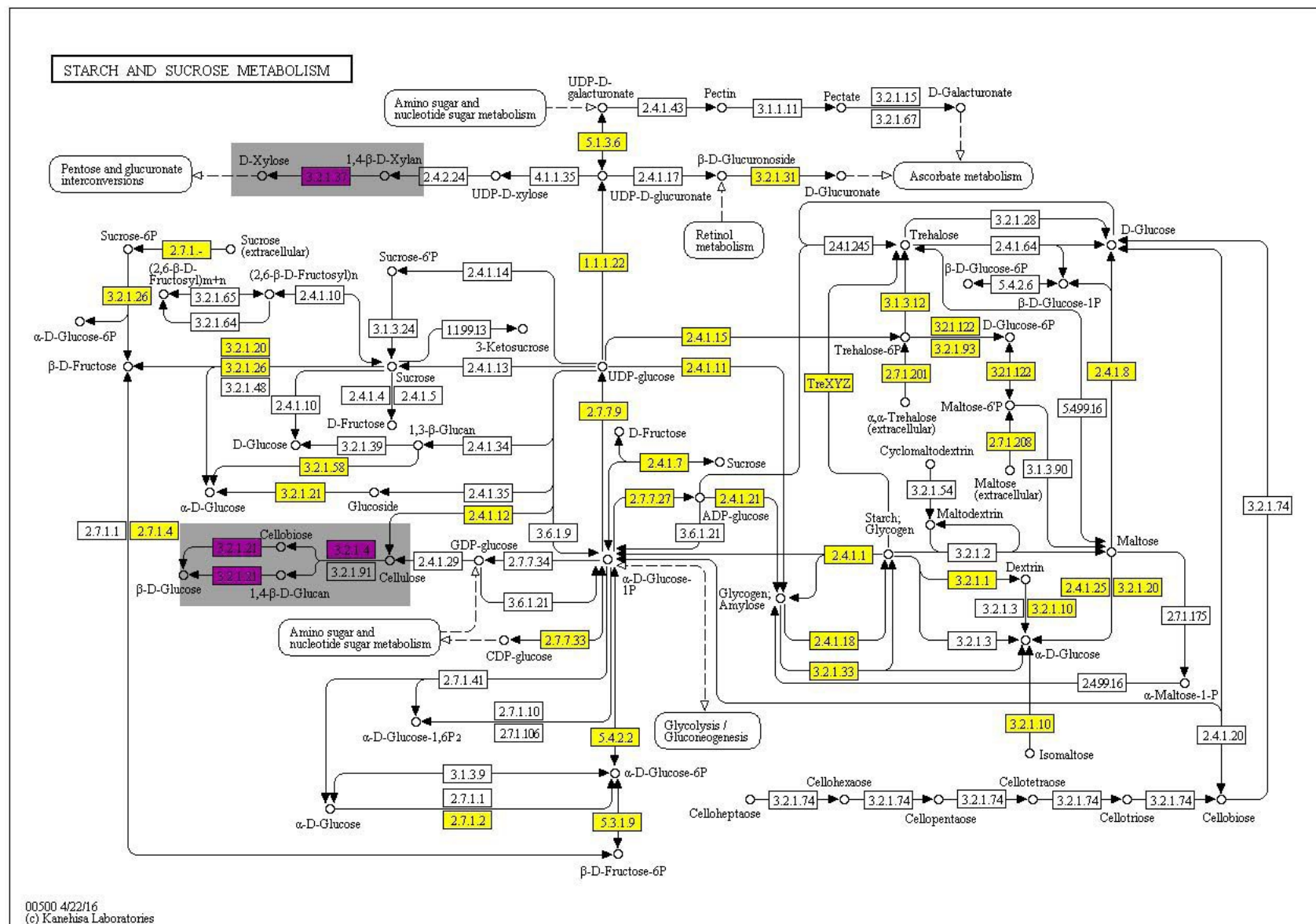

### C Herbivore

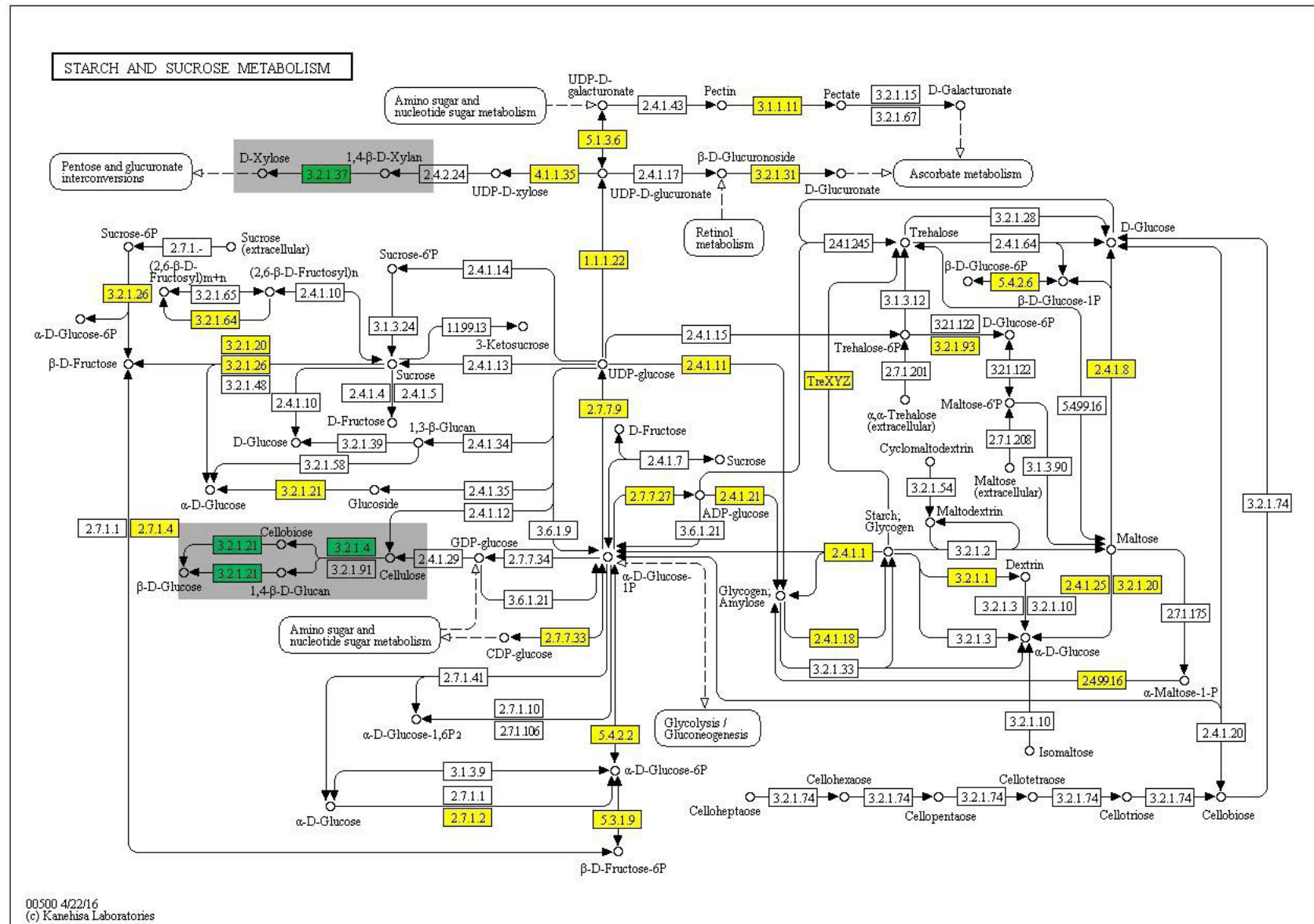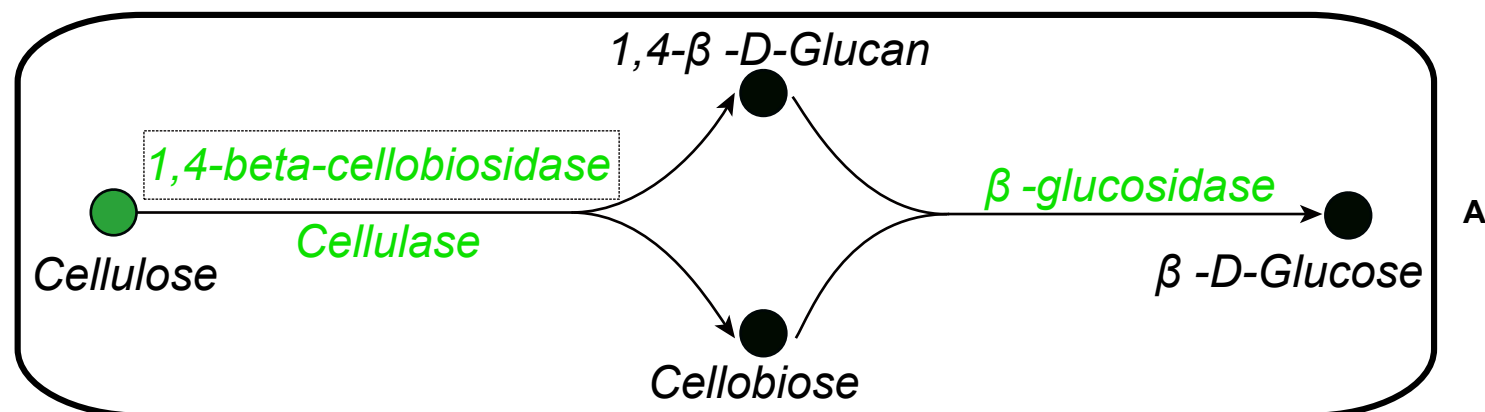

## D Omnivores

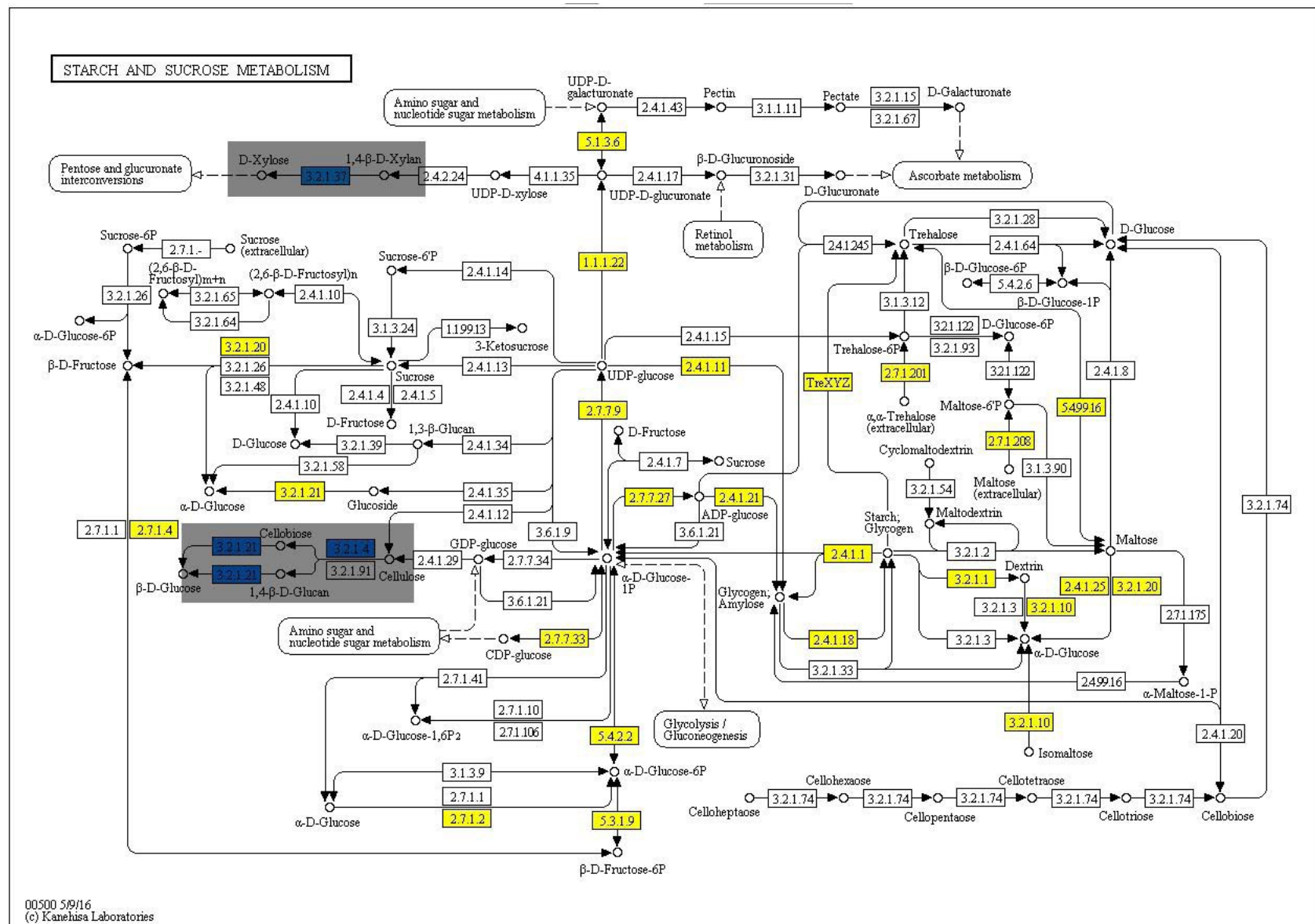

## E Giant pandas

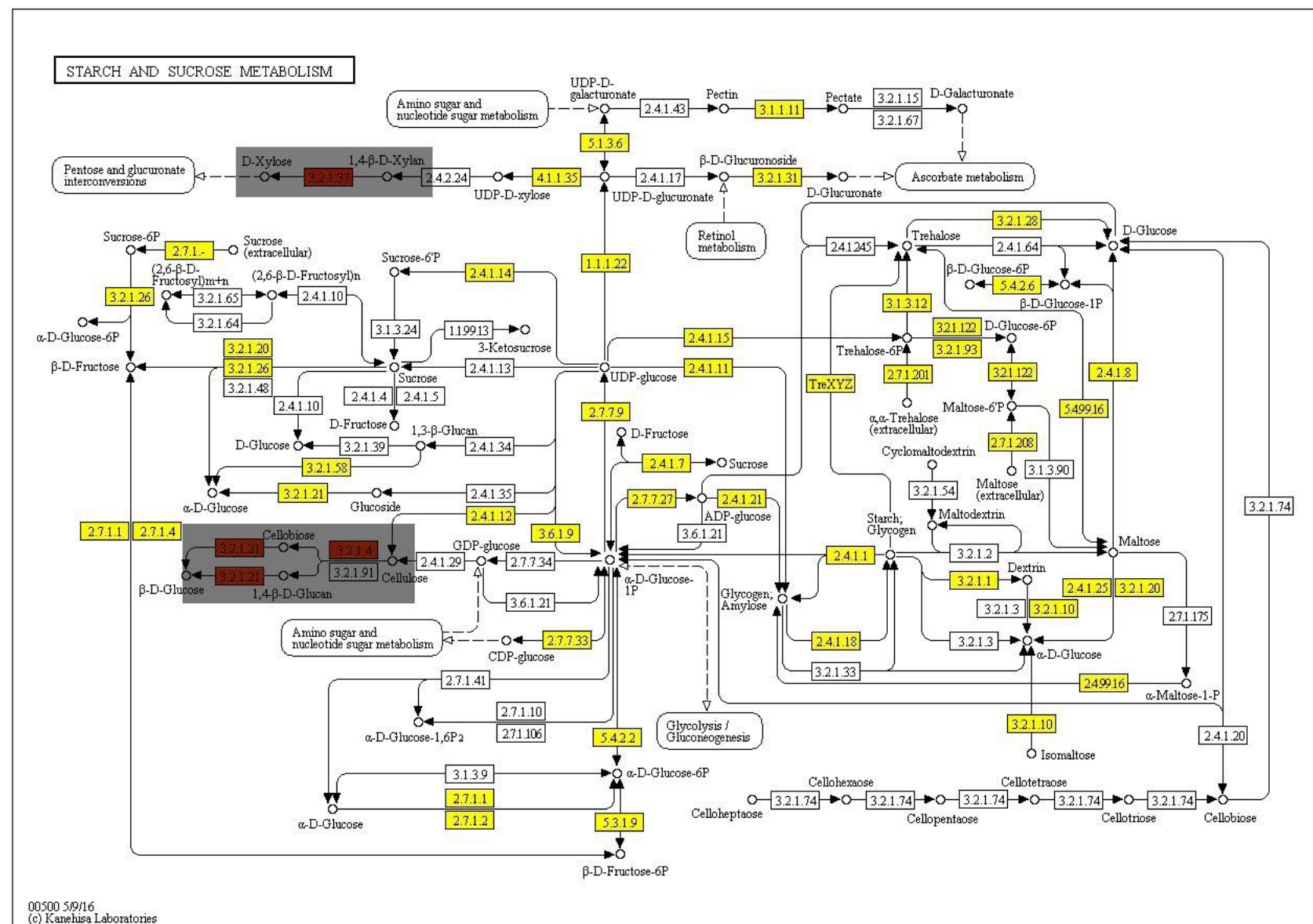

Supplement: Figure S2 — The pathways of cellulose degradation in the gut microbiota of different mammalian dietary categories: (A) Schematic of pathways of cellulose digestion. (B) Carnivores, (C) Herbivores, (D) Omnivores, and (E) Giant panda. All mammalian groups harbor the common pathways for cellulose- and hemicellulose-degradation. [file Image2.PDF]

Figure S3

Fiber-degrading genes

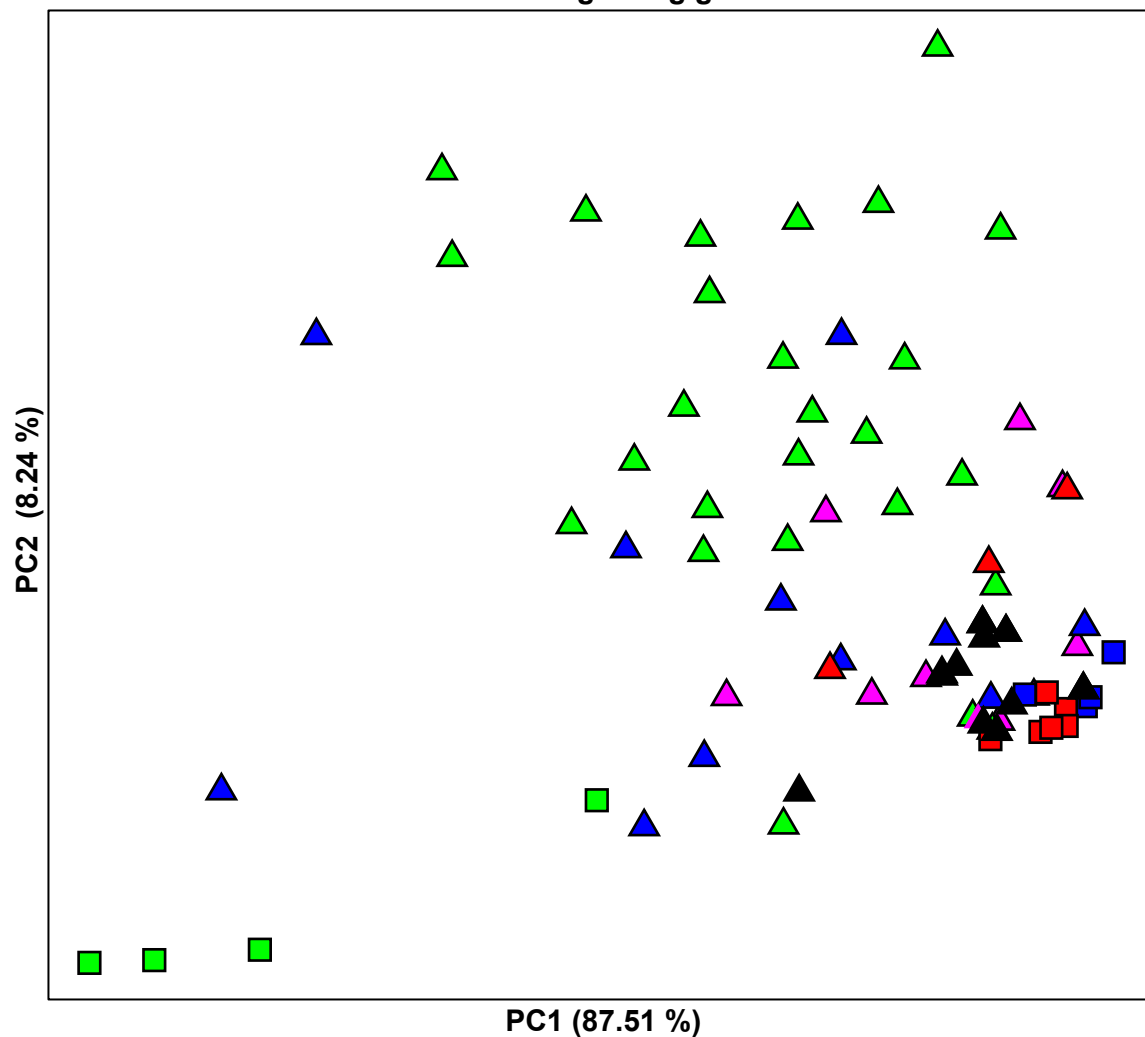

Supplement: Figure S3 — Principal components analysis ordinations of metagenomic function in the gut of giant panda and terrestrial mammals when considering fiber-degrading genes. [file Image3.PDF]

Figure S4

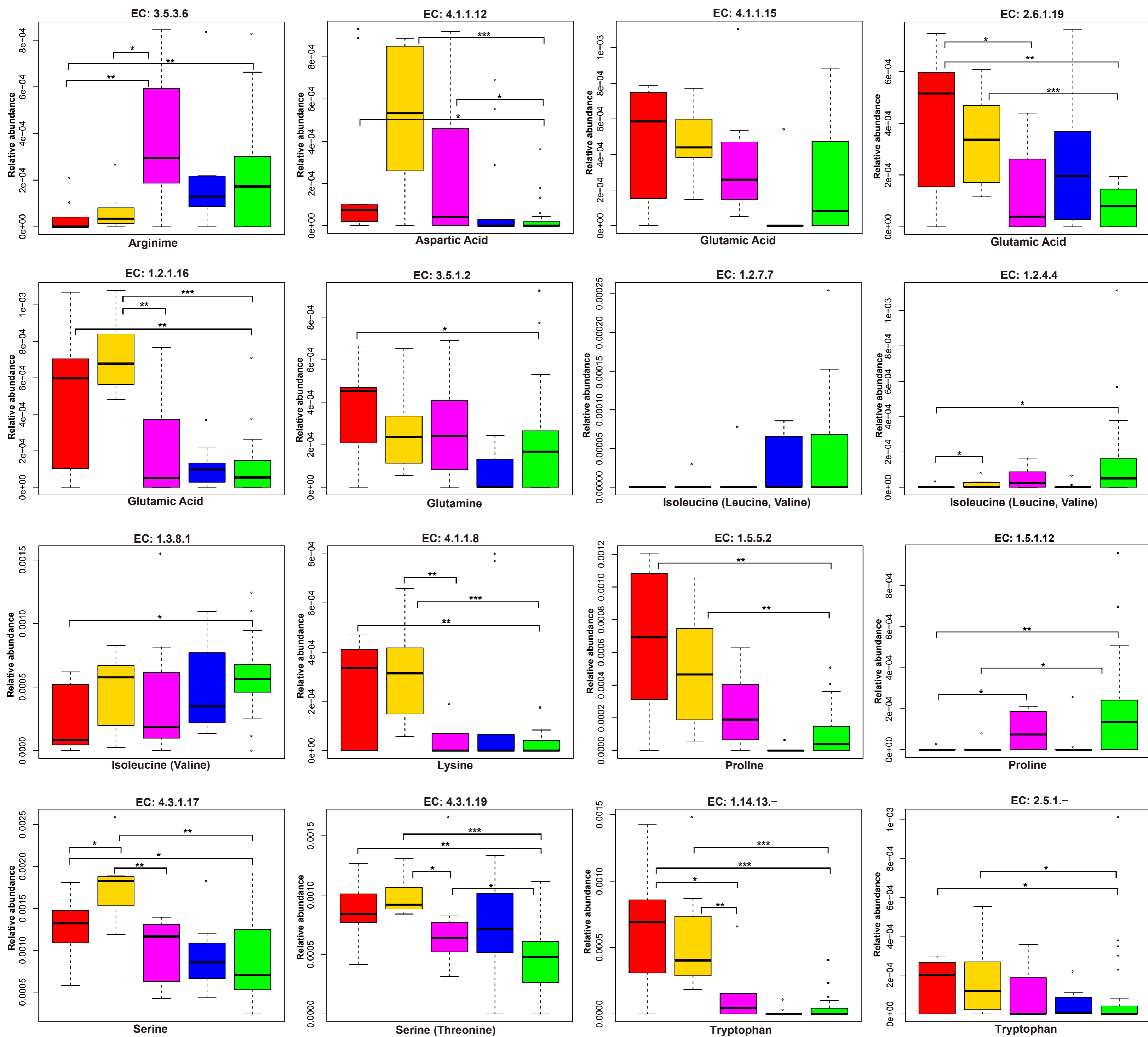

## Groups

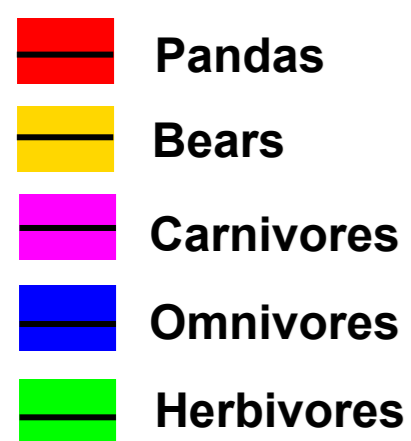

Supplement: Figure S4 — Relative abundance of enzymes involved in amino acid degradation pathways in giant pandas, bears, carnivores, ominivores, and herbivores. Except EC: 3.5.3.6 and EC: 1.5.1.12, the giant pandas reflect the similar result to those of bears and carnivores: most of the enzymes involved in amino acid-degradation pathways were more enriched than herbivores (* < 0.05, ** < 0.01, and *** < 0.001 by Mann-Whitney test). [file Image4.PDF]

Figure S5

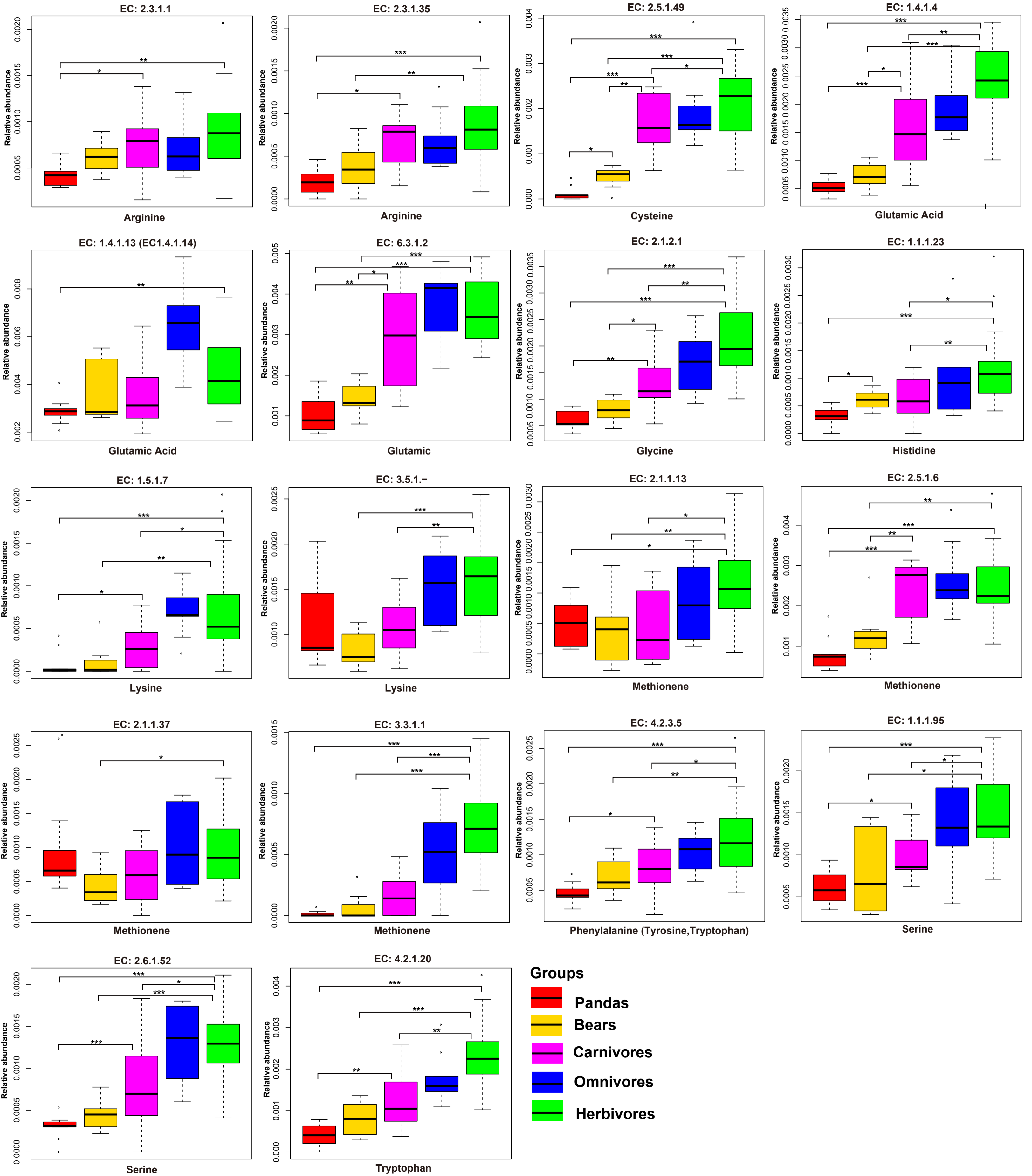

Supplement: Figure S5 — Relative abundance of enzymes involved in amino acid biosynthesis reactions in giant pandas, bears, carnivores, ominivores, and herbivores. The herbivores harbor more proportional abundance of enzymes involved in amino acid biosynthesis than giant pandas, bears, and carnivores (* < 0.05, ** < 0.01, and *** < 0.001 by Mann-Whitney test). [file Image5.PDF]

Figure S6

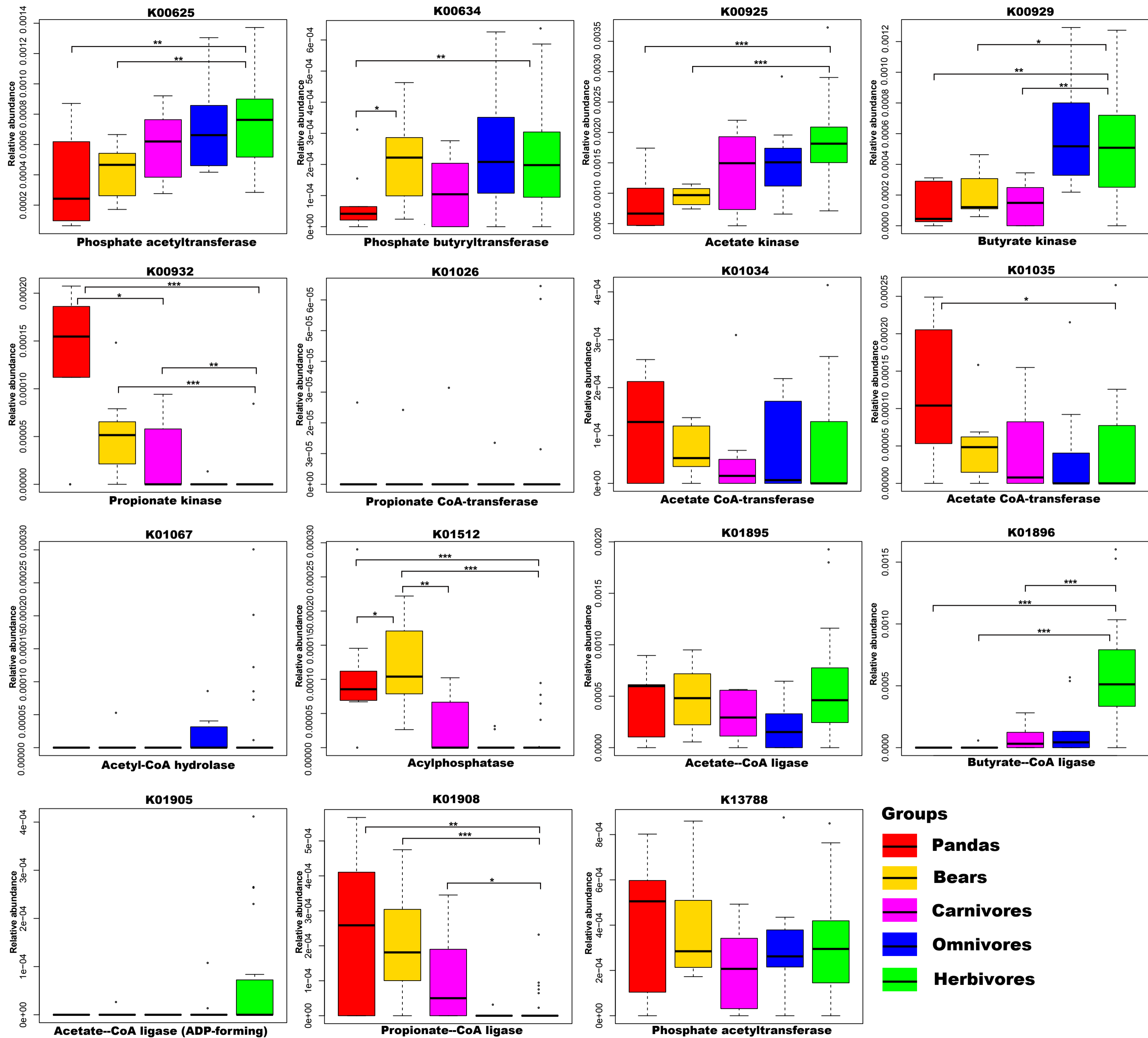

Supplement: Figure S6 — Relative abundance of SCFA-associated enzymes in giant pandas, bears, carnivores, ominivores, and herbivores from the analysis of Sanders et al. (our data reveal the completely consistent results with Sanders et al. (2015) (* < 0.05, ** < 0.01, and *** < 0.001 by Mann-Whitney test). [file Image6.PDF]

Figure S8

A

Giant panda      Herbivore

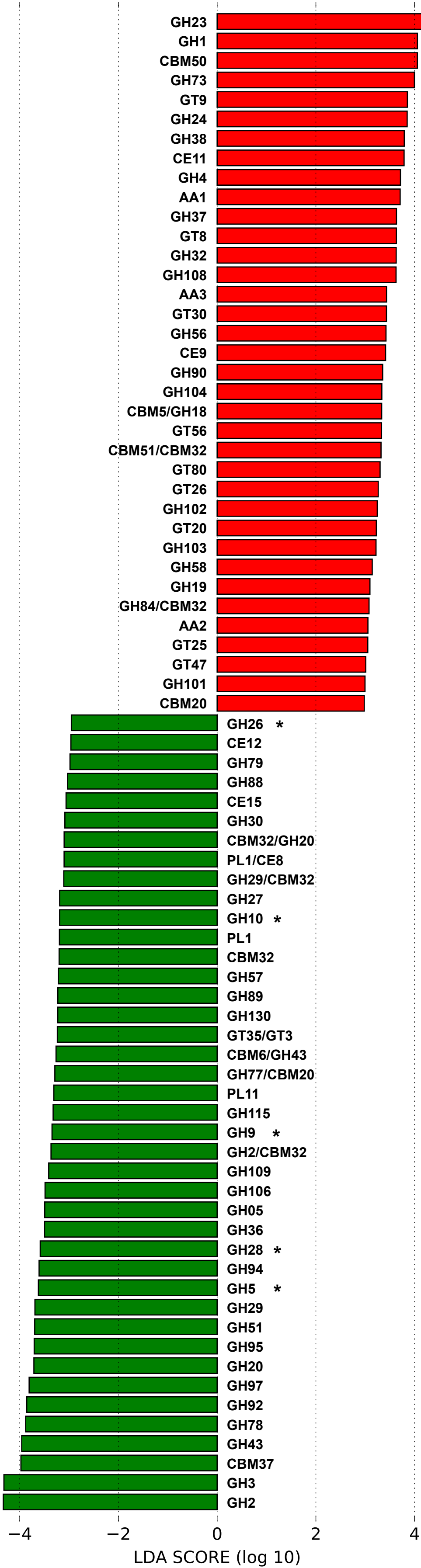

B

Carnivore      Herbivore

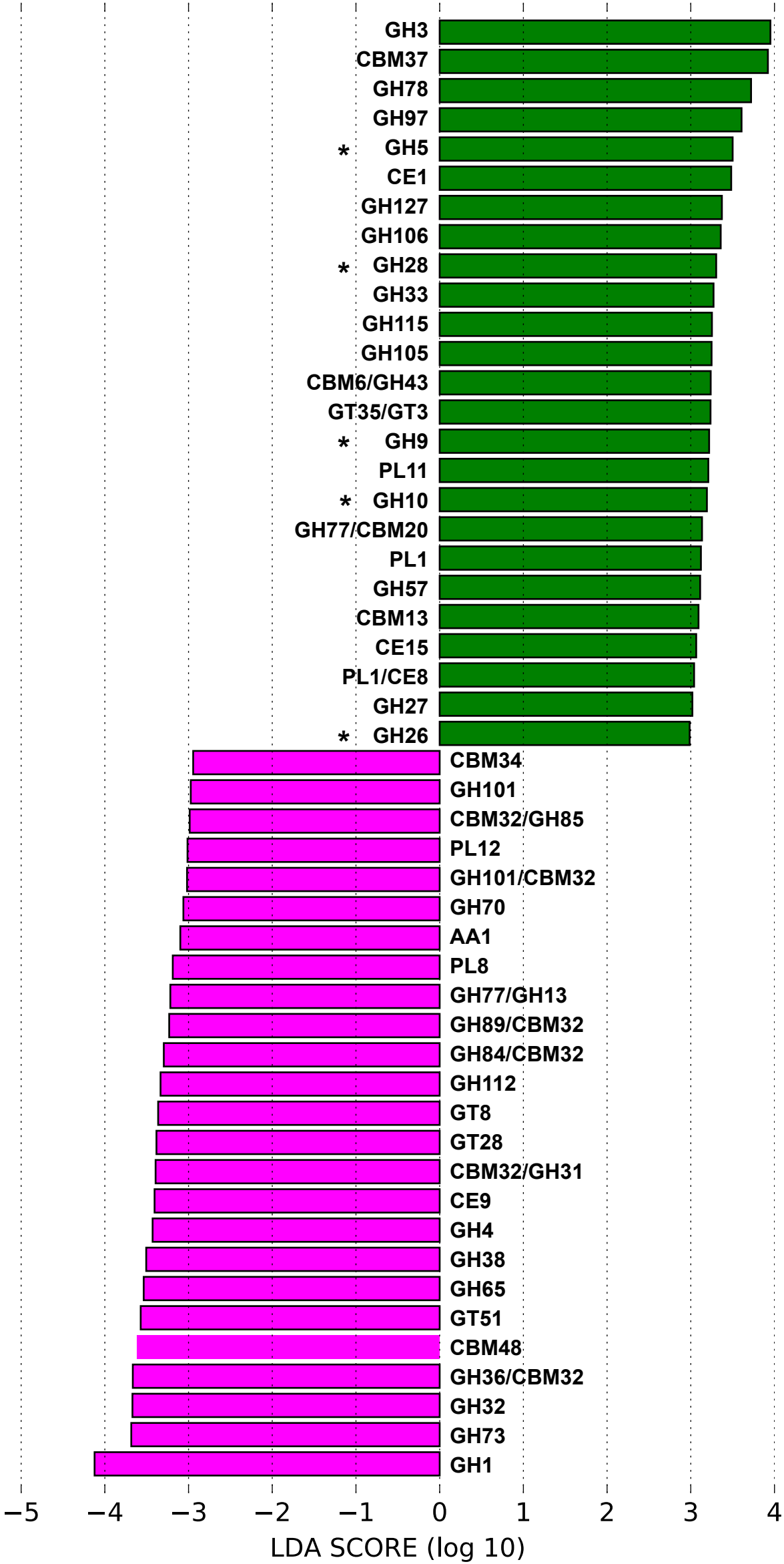

C

Carnivore      Gaint panda

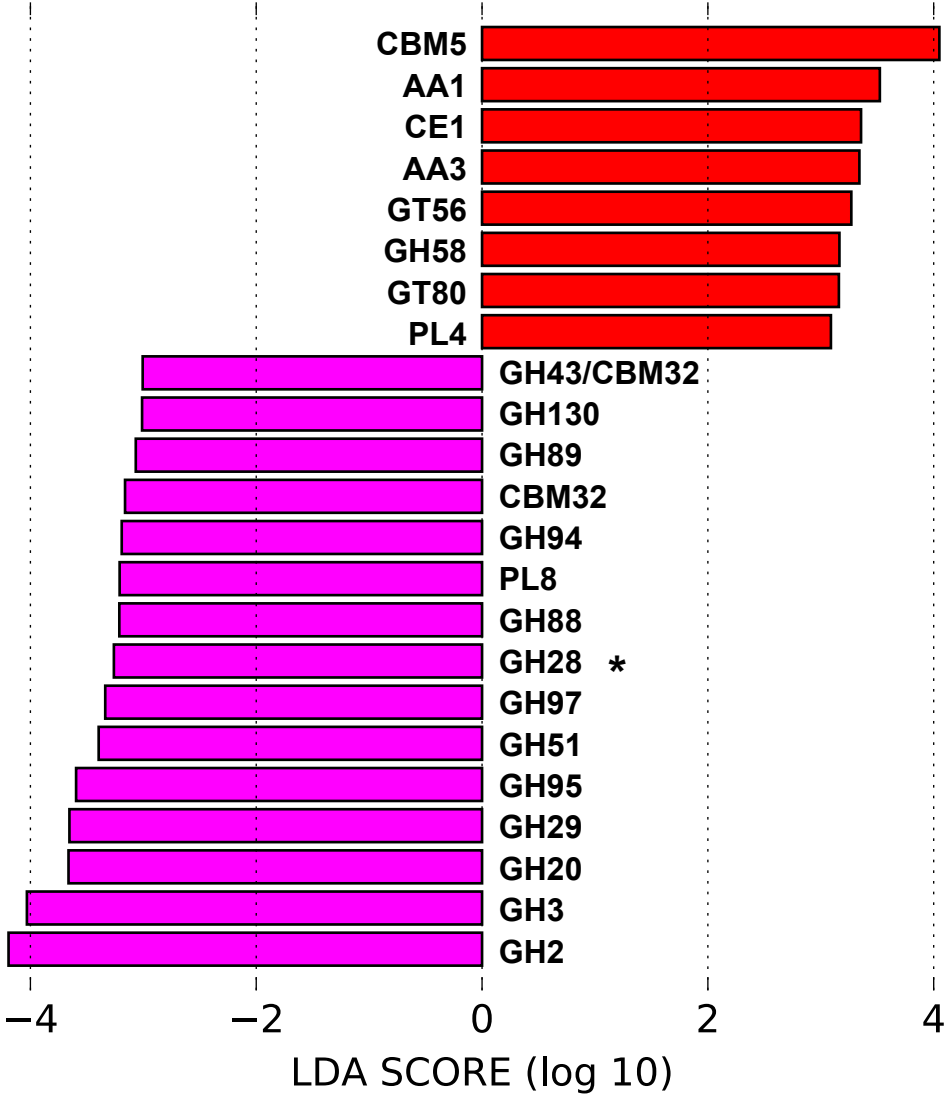

Supplement: Figure S8 — CAZYme families significantly differentiated among giant pandas, herbivores, and carnivores by LEfSe. (P-value cut-off of 0.01 and a minimum effect size of 3, * represent the GH family is correlate with cellulose and hemicellulose degradation). [file Image8.PDF]
