# Supplementary material for: Metagenomic Study Suggests That the Gut Microbiota of the Giant Panda (Ailuropoda melanoleuca) May Not Be Specialized for Fiber Fermentation
Source: Front Microbiol. 2018 Feb 16;9:229. doi: 10.3389/fmicb.2018.00229 (PMC5820910; doi:10.3389/fmicb.2018.00229)
Supplement: Figure S7 — Relative abundance of key enzymes associated with the Wood-Ljungdahl and hydrogenotrophic methanogenesis pathway in giant pandas, bears, carnivores, ominivores, and herbivores in the analysis of Sanders et al. (2015) (our data reveals completely consistent results with Sanders et al., 2015) (* < 0.05, ** < 0.01, and *** < 0.001 by Mann-Whitney test). [file Image7.PDF]

**Figure S7**

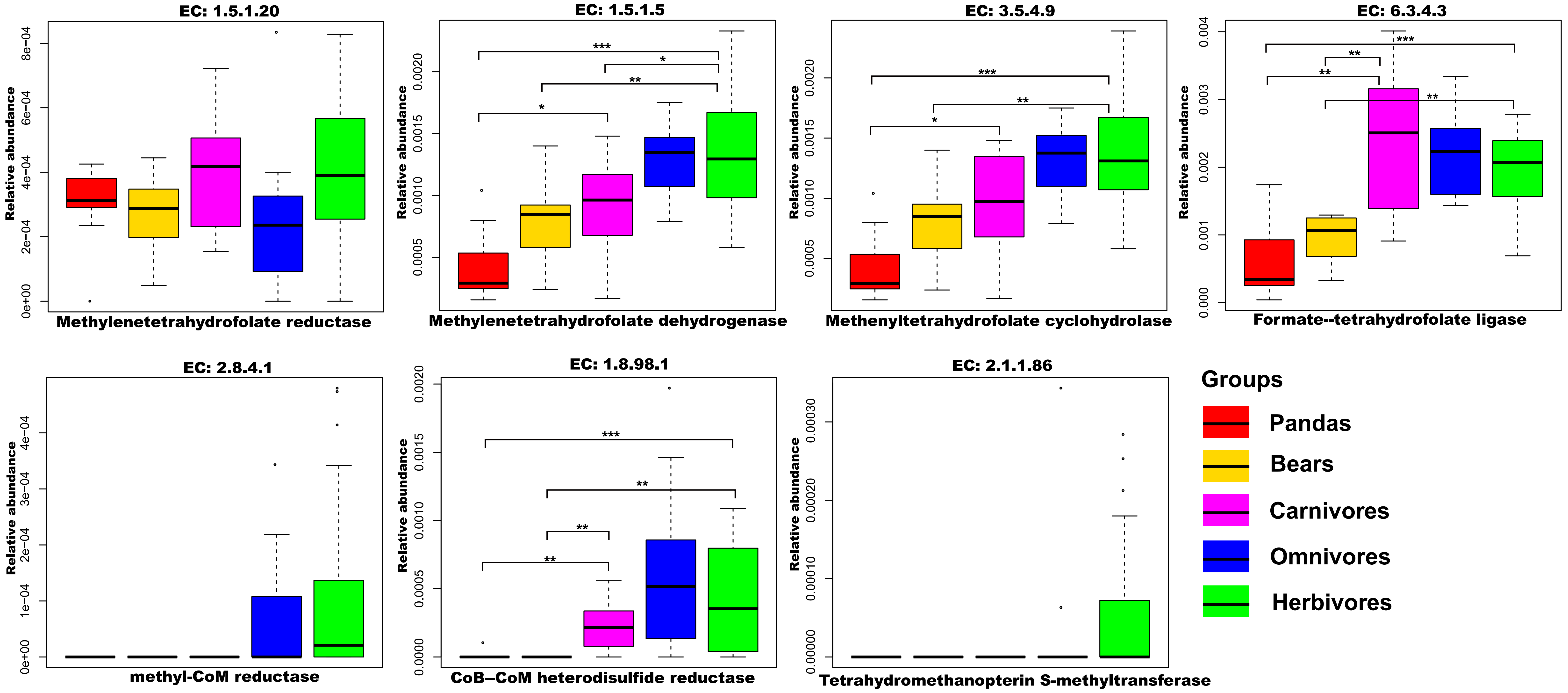
EC: 1.5.1.20
Methylenetetrahydrofolate reductase
EC: 1.5.1.5
Methylenetetrahydrofolate dehydrogenase
EC: 3.5.4.9
Methenyltetrahydrofolate cyclohydrolase
EC: 6.3.4.3
Formate--tetrahydrofolate ligase
EC: 2.8.4.1
methyl-CoM reductase
EC: 1.8.98.1
CoB--CoM heterodisulfide reductase
EC: 2.1.1.86
Tetrahydromethanopterin S-methyltransferase
Relative abundance
Groups
Pandas
Bears
Carnivores
Omnivores
Herbivores
